# The Gb3-synthase A4GALT is an epigenetically regulated driver of tumor invasiveness in gastrointestinal cancer

**DOI:** 10.1186/s12885-026-15600-7

**Published:** 2026-01-27

**Authors:** Noah-David Hirsch, Markus Perl, Simon Holzinger, Christoph Barz, Stefan Enßle, Widya Johannes, Anja Conrad, Jonas J. Unterholzner, Viktoria Obermeier, Markus Tschurtschenthaler, Ludger Johannes, Klaus-Peter Janssen

**Affiliations:** 1 Department of Surgery, TUM University Hospital Rechts der Isar, School of Medicine and Health, Ismaninger Str. 22, Munich, 81675 Germany; 2 Department of Internal Medicine III, Hematology & Medical Oncology, University Hospital Regensburg, Regensburg, Germany; 3 Regensburg Center for Biochemistry (RCB), University of Regensburg, Regensburg, Germany; 4 Leibniz-Institute for Immunotherapy, Regensburg, Germany; 5 Department of Gynecology, University Hospital Würzburg, Würzburg, Germany; 6 Department of Medicine III, TUM University Hospital Rechts der Isar, Munich, Germany; 7 Department of Internal Medicine II, Gastroenterology, LMU University Hospital, Munich, Germany; 8 Biomimetics-Innovation-Centre, City University of Applied Sciences, Bremen, Germany; 9 Department of Internal Medicine I, TUM University Hospital Rechts der Isar, Munich, Germany; 10 Chair of Translational Cancer Research and Inst. of Exp. Cancer Therapy, TUM University Hospital, München, Germany; 11 Center for Translational Cancer Research (TranslaTUM), TUM University Hospital, Munich, Germany; 12 Division of Translational Cancer Research. German Cancer Research Center (DKFZ) and German Cancer Consortium (DKTK), Heidelberg, Germany; 13 Chemical Biology of Cancer Unit, Institut Curie, PSL Research University, U1339 INSERM, UMR3666 CNRS Paris, France; 14 SAIRPICO Team, Inria Center at University of Rennes, U1339 INSERM, Institut Curie, Chemical Biology of Cancer Unit, Paris, France

**Keywords:** Gastrointestinal cancer, Lipid metabolism, Glycosphingolipids, Tumor invasion, Tumor progression

## Abstract

**Background:**

Tumor lipid metabolism has emerged as a critical, yet underexplored, determinant of cancer progression with clinical and prognostic importance. A membrane lipid species of particular interest is globotriaosylceramide (Gb_3_/CD77), a glycosphingolipid that serves as cellular receptor for the bacterial Shiga toxins, and is upregulated in various malignancies. While preclinical studies have suggested a pro-tumorigenic role for Gb_3_, the regulatory drivers of its biosynthesis in human tumors have remained elusive.

**Methods:**

The glycosyltransferase A4GALT, responsible for Gb_3_ biosynthesis, and the degrading enzyme α-galactosidase A (α-GLA) are two essential molecular determinants of Gb_3_ cell surface expression. Expression of these enzymes was analyzed on mRNA level in tissues from colorectal cancer, published datasets from gastric, pancreatic, esophageal and colorectal cancer (1213 patients) and in human cell lines. Pharmacological manipulation in vitro using the hypomethylating agent 5-Aza-2-deoxycytidine and histone deacetylase (HDAC) inhibitors induced A4GALT expression in DLD1 colon cancer cells and Gb_3_ biosynthesis. A4GALT deficiency was induced by CRISPR-Cas9 mutagenesis in human HCT116 colon cancer cells, and its putative effects tested for proliferation, cell migration and invasion.

**Results:**

A4GALT deficiency in HCT116 cells confirmed its essential role for Gb_3_ biosynthesis, leading to resistance against Shiga toxin 1a, and to reduced cancer cell migration and invasion. Gene enrichment analyses revealed that high A4GALT and low α-GLA expression is associated with a distinct gene expression program in gastric, pancreatic and colorectal cancer, including increased signatures of epithelial–mesenchymal transition. A4GALT expression is regulated by chromatin accessibility and DNA methylation at a defined intronic enhancer. In accordance, pan‑cancer analysis of TCGA datasets validated the A4GALT‑high/α‑GLA‑low signature as a negative prognostic indicator across gastrointestinal tumor entities.

**Conclusions:**

Our study uncovers an epigenetically regulated lipid metabolic axis involving A4GALT and Gb_3_ that contributes to aggressive tumor behavior. Of note, this pathway may be therapeutically targetable using natural or synthetic Shiga toxin B-subunit derivatives.

**Supplementary Information:**

The online version contains supplementary material available at 10.1186/s12885-026-15600-7.

## Introduction

Recently, tumor cell metabolism has gained renewed attention as a hallmark of cancer, with a growing body of evidence indicating that metabolic reprogramming is a driver, rather than a by-product, of malignancy [1]. Among these alterations, changes in lipid metabolism, particularly in the composition and function of membrane-associated lipids, have emerged as powerful predictors of tumor behavior and patient outcome. We recently identified a robust, tumor-specific lipidomic signature of triacylglycerols and sphingolipids in colorectal cancer that was consistent across independent patient cohorts and mouse models [2]. These findings highlight the potential of lipidomic profiling as a prognostic tool and raise important questions about the regulatory mechanisms and functional consequences of the altered lipid metabolism in cancer. A membrane glycosphingolipid (GSL) of particular interest is globotriaosylceramide (Gb_3_), also known as CD77. Gb_3_ is accumulated in several gastrointestinal cancers, including colorectal cancer (CRC), gastric cancer (GC) and pancreatic ductal adenocarcinoma (PDAC) [3–6]. Together, these malignancies account for approximately 3.5 million new cancer cases annually and remain responsible for over 2.1 million cancer-related deaths worldwide each year [7]. Beyond gastrointestinal tumors, elevated Gb_3_ levels have also been reported in Burkitt’s lymphoma [8], cervical cancer [9], ovarian cancer [10], breast cancer [11, 12] and osteosarcoma [13]. Of note, Gb_3_ has gained special interest since it can be targeted specifically with the non-toxic B subunit of Shigatoxin (STxB), for example for early tumor detection. Moreover, chemotherapeutic agents were delivered efficiently to Gb_3_-positive cancer cells by coupling to STxB, greatly increasing the therapeutic efficacy and essentially sparing Gb_3_-negative normal cells. Thus, Gb_3_ might be a target for diagnostic and therapeutic applications. The neutral lipid serves as the high-affinity receptor for the bacterial Shiga toxin in the context of hemolytic uremic syndrome (HUS) [14]. The toxin comprises a cytotoxic A-subunit and a pentameric B-subunit (STxB), which binds specifically to Gb_3_ molecules [15]. This interaction triggers lipid reorganization in membrane microdomains and initiates retrograde transport, directing uptake from endosomes to the endoplasmic reticulum (ER) via the Golgi apparatus bypassing lysosomal degradation [16–18]. In pre-clinical models, STxB has been used to deliver imaging and therapeutic payloads. Owing to their stability, efficient cellular uptake, and low immunogenicity, STxB conjugates have demonstrated strong potential for endoscopic, positron emission tomography, and ultrasound imaging in xenograft and genetically engineered mouse models of gastrointestinal cancers [6, 19, 20].

Gb_3_ is synthesized in the Golgi apparatus by the enzyme α1,4-galactosyltransferase (A4GALT), which catalyzes the terminal step of its biosynthesis by transferring a galactose residue to lactosylceramide. Its degradation is initiated in lysosomes by the α-galactosidase A (α-GLA), which removes the terminal galactose moiety [21]. While these enzymatic steps have been well studied in the context of inherited disorders – such as the p blood phenotype caused by A4GALT deficiency [22] and Fabry disease resulting from α-GLA deficiency [23] – the regulatory mechanisms that modulate A4GALT and α-GLA expression in cancer remain largely unexplored.

Although glycosphingolipids are known to regulate membrane organization, receptor clustering, and immune responses, the specific pathophysiological role and regulatory control of Gb_3_ in tumors remains somewhat unclear [21]. While some studies suggest that Gb_3_ promotes tumor progression through enhanced invasion and cell survival [24, 25], others report that A4GALT may play a protective role by suppressing epithelial-to-mesenchymal transition [26, 27]. Additional evidence links Gb_3_ to the regulation of cell death [11, 28] and immune evasion [29], indicating a complex, context-dependent function.

As lipid metabolism gains increasing recognition as a key driver of cancer phenotypes, a deeper understanding of the mechanisms regulating Gb_3_ cell surface expression and its clinical impact is essential to elucidate its role in tumor progression and therapy response.

## Materials and methods

### Patient collective for staining of Gb_3_ cell surface expression

Tissue samples were obtained from patients (*n* = 17) who underwent curative surgical resection at the Department of Surgery, TUM University Hospital Rechts der Isar (Munich, Germany) between 2016 and 2024. Non-malignant and tumor tissues were selected by a trained pathologist based on histological assessment. Samples were immediately snap-frozen in liquid nitrogen and stored at –80 °C. Written informed consent was obtained from all participants. Ethical approval was granted by the Ethics Committee of the TUM School of Medicine and Health (#1926/2007 and #5428/12).

### TCGA patient cohort and bioinformatic analysis

Survival, methylation (Illumina's 450 k methylation arrays) and gene expression data for A4GALT and α-GLA genes were obtained from the GDC portal of TCGA (The Cancer Genome Atlas) [30]. A total of 1.248 patients, 597 cases with colorectal carcinoma (CRC), 354 cases with gastric carcinoma (GC),176 patients with pancreatic carcinoma (PC) and 86 patients with esophageal adenocarcinoma (EAC) were included. Cut-off values were cohort composition and the optimal cut-off values for gene expression were obtained from the Human Protein Atlas, with the cut-off values determined using the maximum log-rank statistic or calculated in R [31]. Based on cut-off values, groups with high and low expression of the respective target gene were defined. Patient survival depending of gene expression was visualized and analyzed by Kaplan–Meier curves and Cox-Regression to investigate the impact of gene expression on survival. Methylation data was visualized in R (v4.4.2 [32]) with karyoploteR (v1.32.0 [33]). Differential gene expression analysis was performed using DESeq2 (v1.46.0 [34]) and visualized with EnhancedVulcano (v1.24.0 [35]). Gene set enrichment analyses were performed with clusterProfiler (v4.14.6 [36, 37]) using the Hallmark Gene Sets (H collection) from the Molecular Signatures Database [38].

### Epigenomic profiling of cell lines

Methylation profiles (Illumina's 450 k methylation arrays) of DLD1 and HCT116 cell lines were obtained from the Gene Expression Omnibus (GEO), accessible under the accession GSE57342 [39]. DLD1 and HCT116 chromatin accessibility data (ATAC-Seq) were also derived from GEO with the accession GSE186589 [40]. Processing was performed using a standard ATAC-Seq pipeline implemented in snakemake (v9.1.2 [41]). In short raw reads were quality filtered and -controlled with fastp (v0.24.0 [42]) before mapping them to the hg19 genome with bowtie2 (v2.5.4 [43]) using "–very-sensitive –no-discordant –no-mixed –no-unal -X 2000" options. Mapped reads were sorted, deduplicated and multimappers removed with samtools (v1.21 [44]) and picard (v3.3.0 [45]). Afterwards only properly paired reads were retained with samtools before shifting all reads and generating coverage tracks using deeptools (v3.5.6 [46]) with “alignmentSieve –ATACshift” and “bamCoverage -bs 10 -normalizeUsing RPKM –extendReads “ options respectively. Epigenomic data was visualized in R (v4.4.2 [32]) using the karyoploteR (v1.32.0 [33]) package utilizing plyranges (v1.26.0 [47]) for data handling.

### Culture of cell lines

All cell lines were tested for Mycoplasma every six weeks, ensuring all experiments were performed with Mycoplasma-free cells. The cell lines used in this study were the gastric cancer lines St3051 [48], AGS (ATCC, CRL-1739), and MKN45 (DSMZ, ACC 409), and the colorectal cancer lines HCT116 (ATCC, CCL-247), DLD1 (ATCC, CCL-221). HCT116, DLD1, St3051, and MKN45 cells were cultured in DMEM supplemented with 7% fetal bovine serum (FBS; Biochrom), 1% penicillin/ streptomycin, and 1% glutamine. AGS cells were cultured in RPMI 1640 medium containing 10% FBS, 1% penicillin/streptomycin, and 1% glutamine. Cells were cultured at 37 °C in a humidified 7% CO_2_ atmosphere. To minimize contamination and prevent phenotypic alterations, cells were stored as frozen stocks and maintained in culture for no longer than six weeks consecutively.

### Reagents and antibodies

The recombinant variant STxB/Cys was produced in an endotoxin-free form using a previously described method [6, 49]. It was covalently linked to the fluorophore Cy3 (Amersham Biosciences) according to the supplier's protocol and established procedures [3]. The study utilized the following antibodies and reagents: DAPI (2-(4-Carbamimidoylphenyl)-1H-indol-6-carboximidamide) (Sigma), BSA-Cy3 (Nanocs). Cell culture reagents were from Invitrogen (Karlsruhe, Germany). 5-Fluorouracil (5-FU) and Oxaliplatin were obtained from the Pharmacy of the TUM Klinikum rechts der Isar (Munich, Germany). STx1a was produced by the Muething lab as described [50].

### Immunofluorescence microscopy

Immunofluorescence microscopy was performed and visualized with a Zeiss AxioObserver Z1 microscope (Carl Zeiss, Jena) as described previously [51]. For the staining of Gb_3_, cryosections fixed with 3% paraformaldehyde were incubated with STxB-Cy3 at a final concentration of 10 μg/mL in PBS containing 0.2% BSA for 30 min. Cells were stained STxB-Cy3 for 1 h at 4 °C. Images were captured using ZEN 3.0 software (Carl Zeiss, Jena). For counterstaining, a specific antibody against von Willebrand factor (Millipore), secondary antibodies from Jackson Immunoresearch (West Grove), and DAPI [2-(4-Carbamimidoylphenyl)-1H-indol-6-carboximidamide] were used (Sigma-Aldrich), and as negative control BSA-Cy3 (Linscott).

### Transwell migration assay

Cell migration was analyzed using transwell assays as described [52, 53]. Corning transwell inserts with an 8.0 μm pore size (Sigma) were pre-coated with medium containing 10% FBS for 1 h at 37°C. Subsequently, 2 × 10^5^ cells were added to each insert and incubated at 37°C for 24 h. After incubation, cells were fixed with 3% paraformaldehyde and stained with 0.1% crystal violet and DAPI. Migrated cells, which adhered to the bottom of the inserts, were visualized using DAPI staining and counted using Zeiss AxioObserver Z1 microscope.

### Wound-healing assay

Cell migration was assessed using a wound-healing assay as described [52, 53]. Cells were pre-treated with 0.625 μg/mL mitomycin C for 2.5 h to inhibit proliferation and seeded (1 × 10^5^/well) in fibronectin-coated (10 μg/mL) 6-well plates in culture-insert chambers (ibidi). After overnight incubation, chambers were removed, cells were washed, and 1 mL medium with 10% FBS was added. Gap closure after 19 h was monitored by phase-contrast microscopy, and wound area was quantified as % of the initial gap using the MRI Wound Healing plugin for ImageJ (v1.54).

### Proliferation and cell viability

Cell proliferation was assessed using the XTT Cell Proliferation Assay Kit II (Sigma) according to the manufacturer’s protocol. Briefly, 2,000 cells/well were seeded in triplicates on two 96-well plates; after 16 h and 48 h, XTT solution was added, and absorbance was measured after 4 h (Mithras LB 940 reader). Proliferation was calculated as the relative absorbance increase between the two time points. For viability assays, 1,000 cells/well were seeded in triplicates, treated for 24 h, and absorbance was measured [52, 53].

### Generation of A4GALT–deficient HCT116 cells via CRISPR/Cas9

A4GALT-deficient cells were created using the CRISPR/Cas9 system. The vector pSpCas9(BB)-2A-Puro (PX459) V2.0 was provided by Feng Zhang (plasmid 62,988; Addgene) [54]. Two guide RNAs (gRNA1: 5′-ATGATCTACTGGCACGTTGT-3′ and gRNA2: 5′-AACGTGCCAGTAGATCATGA-3′) were cloned into the PX459 vector following established protocols. HCT116 cells were transfected with the CRISPR/Cas9 construct using FuGENE HD (Promega) in accordance with the manufacturer’s instructions. Cells underwent selection with 5 μg/mL puromycin for 72 h. Single cell colonies were subsequently isolated for downstream analyses. The CRISPR/Cas9 target regions were amplified using specific primers, and the resulting amplicons were subjected to NGS CRISPR amplicon sequencing (CCIB DNA Core, Massachusetts General Hospital, MA, USA).

### Quantitative real-time PCR

RNA isolation and reverse transcription from both cell lines and tissue samples were conducted using the RNeasy Kit (Qiagen) according to established protocols [55]. cDNA was synthesized using RevertAid H Minus First Strand cDNA Synthesis Kit (Thermo Fisher). Gene expression at the mRNA level was quantified via rt-qPCR, performed on a LightCycler 480 II system (Roche) using the SYBR™ Green Universal Master Mix (BioRad) and specific primers. To normalize the data, hypoxanthine–guanine phosphoribosyl transferase (HPRT) was used as an internal reference transcript. The primers used in the experiments included A4GALT (5′- CTC CTG GTC TGA TCT GGG GA-3′ and 5′- CCC ACA ACG TGC CAG TAG AT-3′), α-GLA [56] (5′- GACTGGCAGAAGCATTGTGTACTC-3′ and 5′- AAAATTTCGCCAGTGATTGC-3′), and HPRT (5′- GCT TTC CTT GGT CAG GCA GTA TAA T-3′ and 5′- AAG GGC ATA TCC TAC AAC AAA CTT G-3′).

### Flow cytometry

Cells (2 × 10^5^) were washed in 1 mL pre-chilled FACS buffer (90% PBS, 10% FCS) and centrifuged at 1500 g for 4 min at 4 °C. Cell pellets were stained with STxB-Cy3 (35 µg/mL, 1:200) or BSA-Cy3 (33 µg/mL, 1:300) by adding 5 µL of each solution, gently mixing, and incubating on ice for 30 min. After washing, cells were resuspended in 500 µL FACS buffer, transferred to FACS tubes, and 5 µL 7-AAD was added. Following a further 30 min incubation on ice, viable cells were analyzed by flow cytometry (FACSCalibur, Becton Dickinson). Doublets, debris, and dead cells were excluded, and histograms were analyzed with CellQuest Pro (Becton Dickinson). BSA-Cy3 served as control for unspecific staining. The threshold for Gb_3_-positive cells was defined using the Gb_3_-negative DLD1 cell line, with < 1% of events positive after STxB-Cy3 staining.

### Statistical analysis

Statistical analyses were performed using GraphPad Prism (v9.5.1), SPSS (v28), and R (v4.4.2). The following packages were used for analysis and visualization in R: tidyverse (v2.0.0 [57]), ggpubr (v0.6.0 [58]), ggprism (v1.0.6 [59]), rstatix (v0.7.2 [60]). Data are presented as mean ± standard deviation unless otherwise stated. Comparisons between two groups were made using Student’s t-test or Mann–Whitney U test, as appropriate. For multiple comparisons, one-way ANOVA was applied. Kaplan–Meier survival curves were analyzed using log-rank tests. A p-value ≤ 0.05 was considered statistically significant. All experiments were independently replicated at least three times (biological replicates).

## Results

### Gb_3_ biosynthesis is correlated with A4GALT expression

**Fig. 1:**
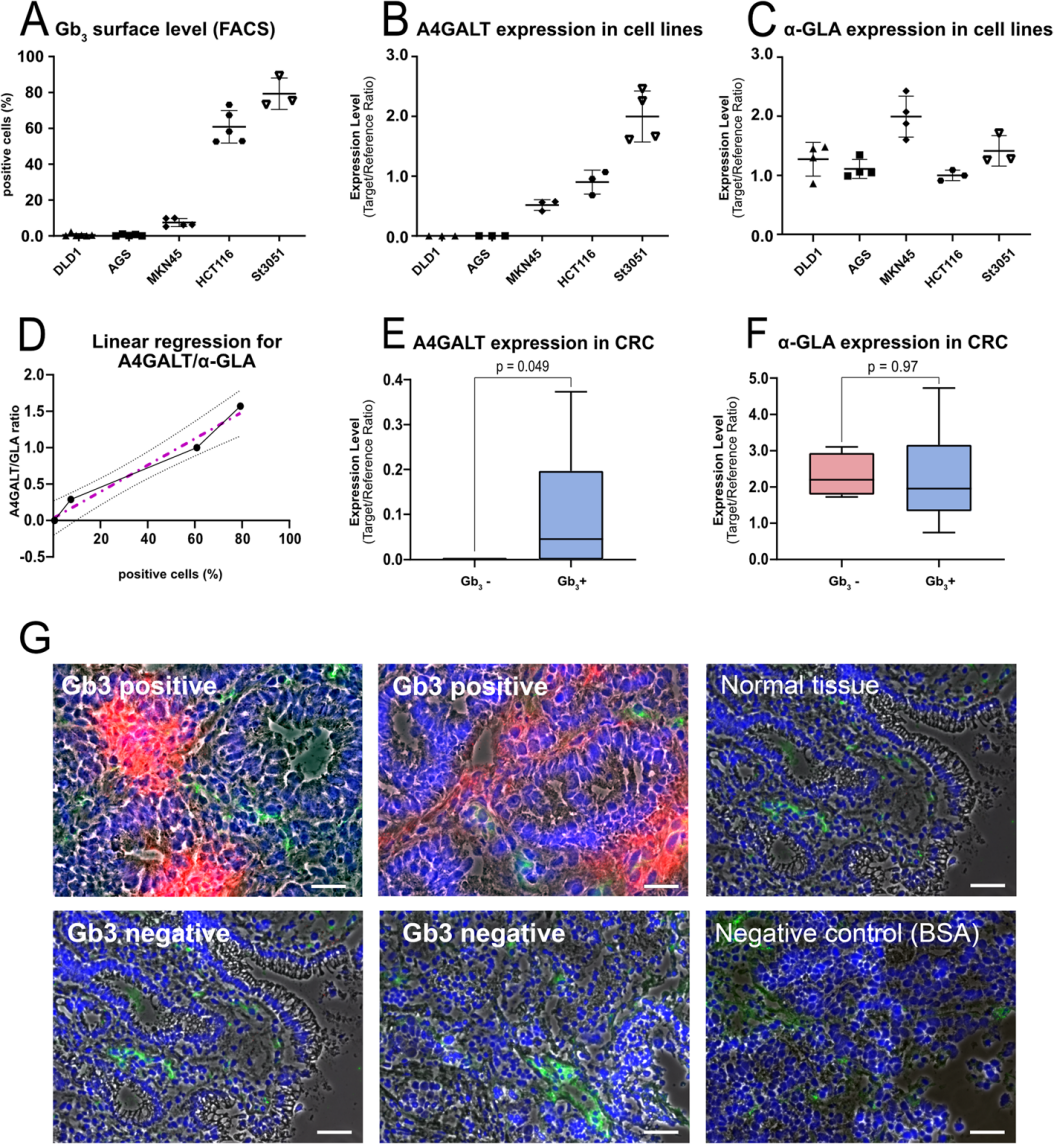
Gb_3_ biosynthesis is correlated with A4GALT expression. **a-c** FACS analysis of Gb_3_ surface levels and rt‑qPCR of A4GALT and α‑GLA in gastric (AGS, MKN45, St3051) and colorectal (DLD1, HCT116) cancer cell lines. Gb_3_ content and A4GALT expression were undetectable in DLD1 and AGS cells, but high in St3051 and HCT116. GLA was expressed in all cell lines at varying levels (presented with mean ± SD; n ≥ 3). **d** Linear regression of the A4GALT/α‑GLA expression ratio vs. Gb_3_ surface levels across all cell lines revealed a strong positive correlation (*p* = 0.0016, R^2^ = 0.98). The dashed magenta line indicates the regression fit and dotted grey lines the 95% confidence interval. **e**,**f** A4GALT and α‑GLA expression in Gb_3_⁺ and Gb_3_⁻ colorectal cancer tissues (classified by STxB staining, *n* = 17 patients). A4GALT expression was significantly higher in Gb_3_⁺ samples (*p* = 0.049), whereas α‑GLA expression did not differ significantly (Tukey box plot). **g** Gb_3_ staining by immunofluorescence microscopy on colon cancer tissue sections. STxB-Cy3 (red) for Gb_3_, anti-vonWillebrand factor antibody for blood vessels (green), and nuclei (DAPI, blue), overlayed with phase contrast images. Two Gb_3_⁺ cases are shown in the upper row, and two Gb_3_⁻ cases in the lower row. As control, normal colon tissue was stained, showing essentially no Gb_3_ detection (upper row, right panel). Control incubation: tumor tissue with BSA-Cy3 instead of STxB-Cy3, with DAPI and anti-vonWillebrand factor (lower row, right panel). Size bars: 25 µM

The relationship between Gb_3_ surface exposure and the mRNA-expression levels of A4GALT and α-GLA was analyzed in gastric (AGS, MKN45, St3051) and colorectal cancer cell lines (DLD1, HCT116) (Fig. 1a-c). Consistent with earlier work [3], DLD1 and AGS cell lines lacked detectable A4GALT expression and were Gb_3_ negative. St3051 and HCT116 exhibited the highest levels of both Gb_3_ exposure and A4GALT mRNA expression. In contrast, α‑GLA was detected in all cell lines, but its expression levels varied between the different cell lines with only MKN45 showing higher levels, which was associated with disproportionally low Gb_3_ surface exposure levels than expected regarding A4GALT expression.

A ratio was calculated from their normalized expression values (A4GALT/ α-GLA) to integrate the impact of both enzymes A4GALT (Gb_3_ synthesis) and α-GLA (Gb_3_ degradation). Linear regression analysis revealed a strong relationship between A4GALT/ α-GLA-ratio and Gb_3_ surface levels (p = 0.0016; R^2^ = 0.9758) (Fig. 1d).

In addition, the cell surface expression of Gb_3_ and expressions of A4GALT and α-GLA were investigated in cryosections of surgically resected tumor samples. Gb_3_ cell surface expression was assessed by fluorescence-coupled STxB staining and classified as negative (Gb_3_–, *n* = 4 patients) or positive (Gb_3_ + , *n* = 13 patients). Only Gb_3_ positivity within the tumor mass was considered for classification. A4GALT mRNA-expression was nearly undetectable in samples with negative Gb_3_ cell surface expression but significantly higher in samples with strong Gb_3_ cell surface expression (*p* = 0.049). α-GLA expression did not differ significantly among these groups (Fig. 1e-f). Illustrative examples for Gb_3_ staining with fluorescently labelled Shigatoxin-B by immunofluorescence microscopy on human colorectal cancer are shown in Fig. 1g. Two positive cases (upper row) and two negative cases are shown, with counterstaining for blood vessels with a specific antibody against vonWillebrand factor. Essentially no Gb_3_ was detectable in non-diseased colon mucosa. As negative control, fluorescently labelled BSA-Cy3 was used at the same concentration as Shigatoxin-B.

### Epigenetic silencing of A4GALT expression and Gb_3_ biosynthesis in CRC cell lines

After identifying A4GALT as a key driver of altered Gb_3_ content, we examined its underlying regulatory mechanisms. Analysis of TCGA cohorts (CRC, GC, PC) revealed no significant genomic alterations, suggesting alternative regulation (Supplementary Fig. 1).

**Fig. 2:**
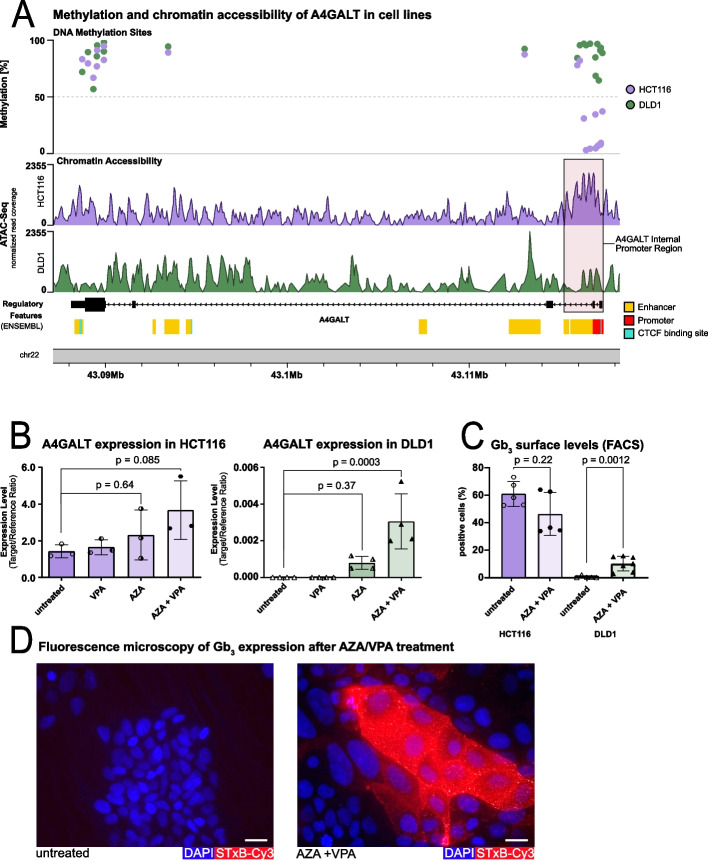
Epigenetic silencing of A4GALT expression blunts Gb_3_ biosynthesis in CRC cell lines. **a** DNA methylation profiles (top, Illumina 450 k methylation array) and Chromatin accessibility (bottom, ATAC-seq) at the A4GALT locus in HCT116 (violet, Gb_3_/A4GALT⁺) and DLD1 (green, Gb_3_/A4GALT⁻). HCT116 cells displayed low promoter methylation and high chromatin accessibility, whereas DLD1 cells showed promoter hypermethylation and reduced accessibility. Color code regulatory features: enhancer (yellow), promoter (red), CTCF‑binding site (turquoise). **b** A4GALT mRNA expression (rt‑qPCR) in DLD1 and HCT116 cells after treatment with Aza, VPA, or sequential Aza/VPA. Sequential treatment induced significant A4GALT upregulation in DLD1 cells (*p* = 0.0003), while HCT116 cells remained unaffected (mean ± SD; n ≥ 3). **c** Gb_3_ surface level (FACS) after epigenetic treatment. Combined Aza/VPA treatment significantly increased Gb_3_ in DLD1 cells (*p* = 0.0079) but had no effect in HCT116 cells (mean ± SD; n ≥ 3). **d** Representative immunofluorescence images of DLD1 cells stained with STxB-Cy3 (red, Gb_3_) and DAPI (blue). Untreated cells lacked detectable Gb_3_, whereas Aza/VPA‑treated cells showed strong membrane‑localized Gb_3_. Scale bar: 20 µm

The A4GALT gene is located on the reverse strand of chromosome 22 (43.09–43.12 Mb, hg19 assembly), with an internal promoter region and several enhancer elements clustered around 43.11 Mb [61]. We analyzed chromatin accessibility and DNA methylation at the A4GALT locus using ATAC-seq and Illumina sequencing data from DLD1 (Gb_3_/A4GALT-) and HCT116 (Gb_3_/A4GALT +) cell lines. In HCT116 cells, the A4GALT enhancer region exhibited low DNA methylation levels and high chromatin accessibility, whereas in DLD1 cells it was highly methylated and showed reduced chromatin accessibility (Fig. 2a). These findings suggest that enhancer hypermethylation and chromatin compaction contribute to transcriptional silencing of A4GALT in DLD1 cells, while hypomethylation and an open chromatin configuration in HCT116 cells permit active A4GALT expression.

To test this hypothesis, DLD1 and HCT116 were treated with the DNA methyltransferase inhibitor 5-Aza-2-deoxycytidine (Aza) and the histone deacetylase inhibitor valproic acid (VPA). In DLD1 treatment with Aza alone resulted in re-expression of A4GALT, while VPA as a single treatment was ineffective. Sequential treatment with Aza followed by VPA exhibited a cooperative effect, further enhancing A4GALT expression (*p* = 0.0003). In contrast, A4GALT-positive HCT116 showed only minor increases in expression after treatment (Fig. 2b) The treatment induced robust re-expression of Gb_3_ at the cell surface in DLD1 cells (*p* = 0.0079), as measured by flow cytometry, whereas no significant changes were observed in HCT116 cells (Fig. 2c). In support, Fig. 2d demonstrates clear Gb_3_ re-expression in treated DLD1 cells, as evidenced by STxB-Cy3 uptake in immunofluorescence microscopy.

### A4GALT promotes migration and invasion of colorectal cancer cells

**Fig. 3:**
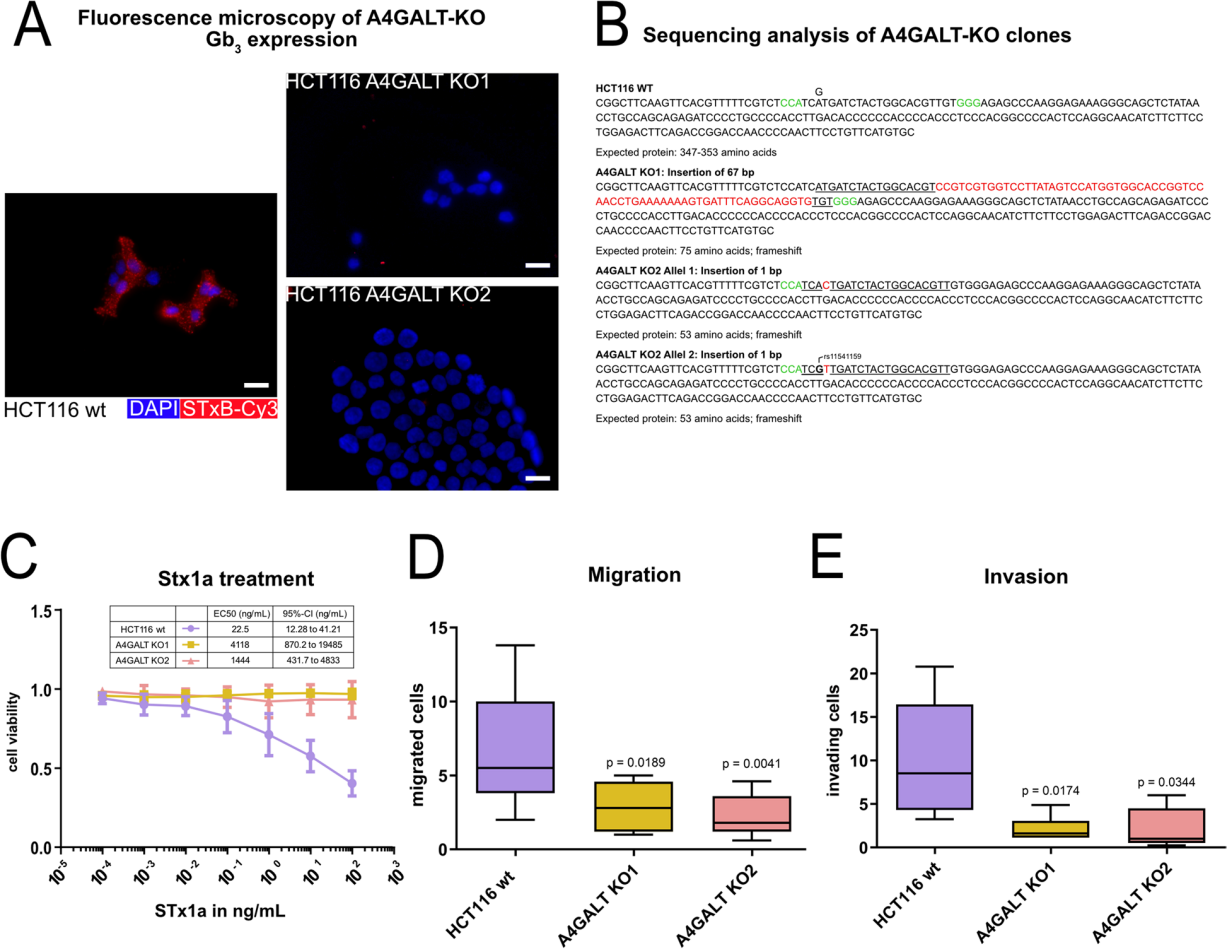
A4GALT promotes migration and invasion in colorectal cancer cells. **a** Representative immunofluorescence images of wildtype and A4GALT‑knockout (KO) HCT116 cells stained with STxB-Cy3 (red, Gb_3_) and DAPI (blue). KO cells showed a complete absence of detectable Gb_3_. Scale bar: 20 µm. **b** NGS analysis of KO clones. KO1 carried a 67‑bp insertion causing a frameshift, while KO2 harbored single‑base insertions also resulting in frameshift mutations. The parental HCT116 line carried the benign variant rs11541159 (c.109A > G; p.Met37Val). PAM sequences are shown in green, gRNA sites underlined, and insertions in red. **c** Dose–response curves to Shiga toxin 1a (Stx1a) treatment. Both KO clones showed significantly higher EC_50_ values compared to parental wildtype cells. (mean ± SD; n ≥ 3). **d** Cell migration assays showed reduced migration in both KO clones compared with wild‑type cells (mean ± SD; *p* = 0.031; n ≥ 4)** e** Matrigel invasion assays revealed a reduction in invasive capacity in KO clones compared with wild‑type cells (mean ± SD; *p* = 0.011; n ≥ 6)

To elucidate the functional role of A4GALT and Gb_3_ in colorectal cancer cells, we generated A4GALT-deficient HCT116 cell lines using CRISPR/Cas9 and investigated their impact on cell behavior. Two independent monoclonal A4GALT-deficient clones (KO1 and KO2) were established through targeted mutagenesis of exon 2 of A4GALT, as confirmed by next-generation sequencing. Both knockout clones exhibited a marked reduction in Gb_3_ surface levels as demonstrated by fluorescence microscopy and flow cytometry (Fig. 3a, Supplementary Fig. 2). KO1 harbored a 67 bp insertion resulting in a frameshift mutation, whereas KO2 carried single‑base insertions in each allele, both leading to frameshift mutations. Sequencing also revealed that HCT116 wildtype (wt) carries two different A4GALT alleles. The observed guanine-to-adenine substitution corresponds to rs11541159 (c.109A > G; p.Met37Val), which is cataloged in UniProt as a fully functional variant [62] (Fig. 3b). For the subsequent functional experiments, data from KO1 and KO2 were pooled.

Functionally, A4GALT-KO cells were highly resistant to Shiga toxin 1a (STx1a), with markedly increased EC50 values of KO (2165 ng/ml; 95% CI: 839.1–5589) versus the wildtype (22.5 ng/ml; 95% CI: 12.28–41.21) (Fig. 3c). Neither cell proliferation nor chemoresistance in vitro to conventional chemotherapeutic agents was significantly affected by A4GALT deficiency (Supplementary Fig. 2). However, cell migration assessed by a 2D wound healing assay was significantly reduced in the A4GALT-deficient clones compared to the wildtype cells (p = 0.031). Furthermore, transwell migration and invasion assays revealed a significant reduction in the invasive capacity of A4GALT-deficient clones (*p* = 0.011) (Fig. 3D-E). Of note, direct quantification of Gb_3_ and other lipid species by mass spectrometry corroborated the findings by indirect Gb3-staining by STxB for both A4GALT-deficient lines, in comparison to parental HCT116 cells. Essentially no Gb_3_ or Gb_4_ (globotetraosylceramide) were detectable in both KO lines, as well as in DLD1 cells which do not express A4GALT. The precusor lipid species ceramide, as starting point for all glycosphingolipids of the globo-series, as well as glucosylceramide and lactosylceramide, were detectable in both A4GALT-KO lines and in DLD1 cells, at similar levels as in parental HCT116 cells (Supplementary Fig. 3).

### Upregulation of tumor promoting genes under Gb_3_ high conditions and epigenetic regulation of A4GALT in tumor patients

**Fig. 4:**
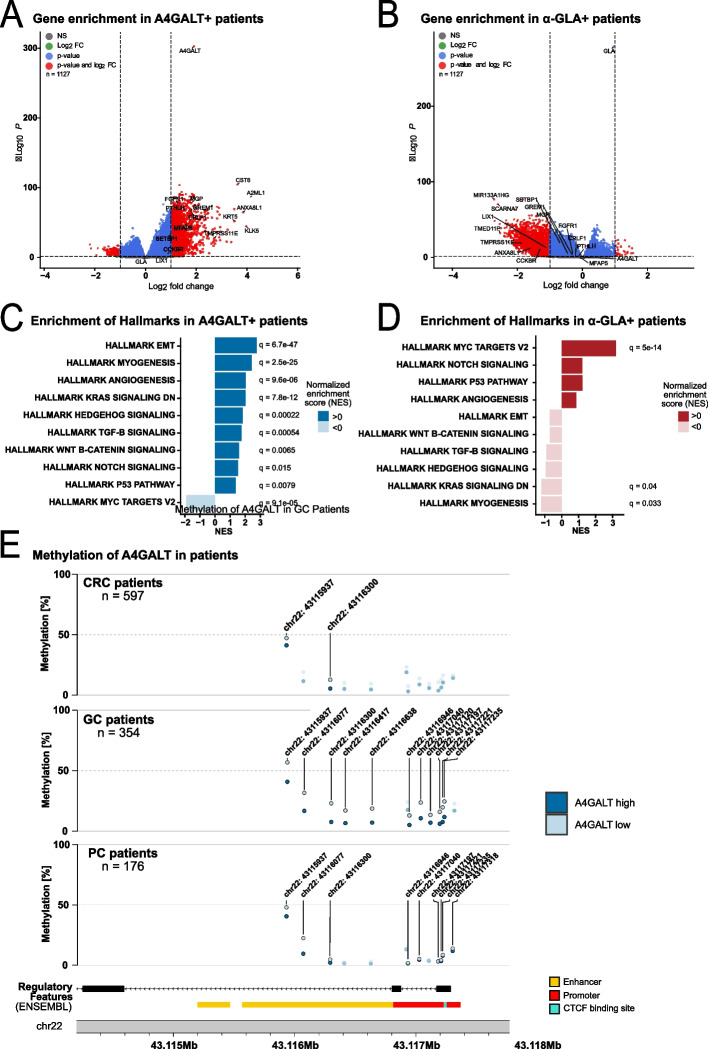
Upregulation of tumor promoting genes under Gb_3_ high conditions and epigenetic regulation of A4GALT in tumor patients. **a–b** Volcano plots of differential expression analysis between high‑ and low‑expression groups for A4GALT **a** and α‑GLA **b**. Significantly upregulated (red) and downregulated (blue) genes are highlighted based on adjusted *p*‑value (*p* < 0.05) and fold‑change thresholds (log2 fold change > 1); non‑significant genes are shown in grey or green. Selected key genes are labeled. **c-d** Hallmark gene set enrichment analysis for A4GALT **c** and α‑GLA **d** expression groups. Asterisks indicate Hallmark sets that were significantly differentially regulated (*p* < 0.05). **e** DNA methylation profiles (Illumina 450 k methylation array) at the A4GALT locus in CRC and GC patients with high or low A4GALT expression. Significantly altered CpG islands are highlighted in color and labeled by identifier, whereas non‑significant sites are faded. Color code regulatory features: enhancer regions (yellow), promoter regions (red), CTCF‑binding sites (turquoise)

To extend our *in-vitro* findings to patient samples, we analyzed RNA-seq data from TCGA cohorts of CRC, GC, and PC. Patients were stratified by A4GALT and α-GLA expression (high vs. low), and differential gene expression analysis was performed. Volcano plots revealed numerous differentially expressed genes in both groups, with A4GALT and α-GLA among the top hits. Several genes including the Annexin A8 Like 1 (ANXA8L1), Cholecystokinin B Receptor (CCKBR), Transmembrane Protease, Serine 11E (TMPRSS11E) and SET Binding Protein 1 (SETBP1) genes were enriched in the A4GALT-high group and showed opposite expression in the α-GLA-high group (Fig. 4a-b). Similar signatures were observed in tumor-specific subset analyses (Supplementary Fig. 4). A4GALT-high tumors showed significantly increased hallmarks [38] of epithelial-mesenchymal transition (EMT), myogenesis, Transforming Growth Factor (TGF)-β, hedgehog, and Wingless-Int1 (WNT)-β-catenin signaling, alongside downregulation of MYC proto-oncogene (MYC) targets. The inverse pattern was observed in α-GLA-high tumors; however, these changes were only partially significant and in part a non-significant trend (Fig. 4c-d).

Detailed analysis of all hallmarks and subset analysis is shown in the Supplementary Fig. 4. Based on the cell line methylation analyses described above, we next analyzed DNA methylation at the A4GALT locus in CRC, GC, and PC cohorts from TCGA. Multiple CpG sites showed significant hypermethylation in the low A4GALT expression groups compared to the high A4GALT expression groups, with similar patterns observed in CRC and GC and less extensive differences in PC. Notably, cg09744051 was consistently hypermethylated in low-A4GALT tumors across all cancer types (Fig. 4e-f). Detailed methylation of all CpG-sites analyzed is presented in the Supplementary Fig. 5.

### Gb_3_ expression is associated with poor patient survival in GI cancer

Functional assays using A4GALT knockout models demonstrated reduced invasiveness, and transcriptomic analyses revealed that high A4GALT and low α-GLA expressions are associated with cancer-promoting pathways and a more invasive phenotype. Next, we analyzed wether Gb_3_ was also expressed on the surface of primary patient derived organoids from patients with colorectal cancer (*n* = 7). Indeed, we could detect Gb_3_ expression by STxB-staining on three of the tested organoids, with a tendency to locally advanced tumors stages, but no obvious association with microsatellite status (Supplementary Fig. 6*)*. We therefore assessed the impact of A4GALT and α-GLA expression on patient survival across GI cancers.

**Fig. 5:**
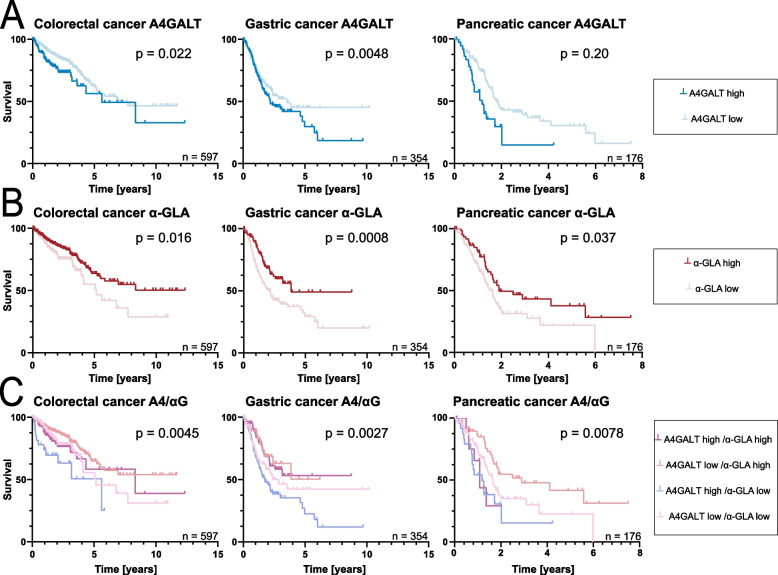
Conditions favouring high Gb_3_ levels associate with poor survival in GI cancer. **a** Kaplan–Meier survival curves for patients with colorectal carcinoma (CRC, *n* = 597), gastric carcinoma (GC, *n* = 354), and pancreatic carcinoma (PC, *n* = 177) stratified into high and low expression groups for A4GALT. Low A4GALT expression was significantly associated with improved survival in CRC (*p* = 0.022) and PC (*p* = 0.0048), with a non-significant trend in GC (*p* = 0.20). **b** Kaplan–Meier survival curves based on α-GLA expression in the same cancer types and cohorts. High α-GLA expression conferred a significant or highly significant survival advantage (CRC: *p* = 0.016; GC: *p* = 0.0008; PC: *p* = 0.037). **c** Combined expression analysis of A4GALT and α-GLA (A4GALT + /α-GLA + , A4GALT + /α-GLA-, A4GALT-/α-GLA + , A4GALT-/α-GLA-) in CRC, GC, and PC. The A4GALT-/α-GLA + group consistently showed a significant survival advantage over the A4GALT + /α-GLA- group (CRC: *p* = 0.0045; GC: *p* = 0.0027; PC: *p* = 0.0078)

Kaplan–Meier analysis showed that high A4GALT expression was associated with poor survival in CRC (*p* = 0.022I) with HR 1.7 (95% confidence interval: 1.1 to 2.7) and PC (*p* = 0.0048), HR 2.3 (95%-CI: 1.3 to 4.1), and showed a non-favorable, though non-significant trend in GC (*p* = 0.20), HR 1.3 (95%-CI: 0.89 to 1.8) (Fig. 5a). Low α-GLA expression correlated with a significant survival disadvantage in all tumor types (CRC: *p* = 0.016, HR 0.60 (95%-CI: 0.40 to 0.91); GC: *p* = 0.0008, HR 0.57 (95%-CI: 0.41 to 0.79); PC: *p* = 0.037, HR 0.64 (95%-CI: 0.42 to 0.98) (Fig. 5b).

Subsequently, combined expression profiles were analyzed, categorizing patients into four groups based on parallel high or low expression (A4GALT + /α-GLA + ; A4GALT-/α-GLA-) and opposing expression (A4GALT + /α-GLA-; A4GALT-/α-GLA +) (Fig. 5c). For all tumor types, survival analysis showed significant survival differences (CRC: *p* = 0.0045; GC: *p* = 0.0027; PC: *p* = 0.0078). The A4GALT + /α-GLA + and A4GALT-/α-GLA- groups exhibited intermediate survival outcomes. However, A4GALT + /α-GLA- exhibited significantly worse survival compared to all other groups (CRC: *p* = 0.0046, HR 2.46 (95%-CI interval: 1.32 to 4.57); GC: *p* < 0.0001, HR 1.78 (95%-CI interval: 1.28 to 2.25); PC: *p* = 0.024; HR 1.87 (95%-CI interval: 1.09 to 3.22). Detailed information on Cox regression results of GC and PC, progression-free and disease-free survival analysis is provided in the Supplementary Fig. 7–9. Of note, analysis of single-cell RNA sequencing data supported an expression of A4GALT in tumor cells of epithelial origin, as well as in some tumor stroma cell types, whereas GLA was more broadly expressed (Supplementary Fig. 10).

Moreover, 86 patients with esophageal adenocarcinoma (EAC) from the TCGA platform were tested. In contrast to the other GI cancer entities, low A4GALT and high GLA expression, respectively, were significantly associated with worse survival. Cell lines from esophageal cancer expressed low or no Gb_3_ at their surface (Supplementary Fig. 11).

## Discussion

Dysregulated lipid metabolism has emerged as a defining hallmark of cancer, yet the specific functional consequences of individual lipid species and their therapeutic relevance remain incompletely understood. Among GSLs, Gb_3_ stands out due to its unique properties: it is tightly enzymatically regulated, functionally implicated in key malignant features, directly accessible on the cell surface, and targetable by multiple therapeutic modalities [20, 21]. Our comprehensive analysis establishes Gb_3_ as a model system for how altered lipid metabolism drives tumor biology in gastrointestinal cancers simultaneously offering opportunities for prognostic stratification and therapeutic intervention.

Gb_3_ is synthesized by A4GALT and degraded by α-GLA, and our data demonstrates that the A4GALT/α-GLA mRNA ratio directly determines the Gb_3_ surface level in vitro. We observed a rather uniform expression of GLA. This may be due to the fact that GLA has other substrates than Gb_3_ and constitutes an essential part of lysosomes, which are critical even for cancer cells. Importantly, α-GLA can influence Gb_3_ levels only when A4GALT is present to initiate its synthesis. This enzymatic antagonism is distinct from the broader effects of glucosylceramide synthase (GCS), which impacts total GSL content but cannot explain the selective accumulation of Gb_3_ [63]. The functional importance of the enzymatic balance between A4GALT and α-GLA is further underscored by Fabry disease, in which α-GLA deficiency results in pathological Gb_3_ accumulation [23].

Although various factors, such as substrate availability and enzyme localization affect cellular Gb_3_-levels [64], these alone do not explain Gb_3_ upregulation in various tumor entities. In particular, the regulation of A4GALT itself remains poorly understood.

Interestingly, we found no significant genetic alterations in A4GALT in patient samples, which prompted us to examine possible epigenetic mechanisms that play a key role in cancer development and may also regulate A4GALT expression [65]. Our data from DLD1 and HCT116 cells underscores that A4GALT expression is repressed via previously described intronic enhancer [61] hypermethylation and reduced chromatin accessibility. Demethylating treatment restored A4GALT expression and induced Gb_3_ surface exposure. Consistent with this, our analysis of patient datasets revealed that specific A4GALT methylation patterns were associated with its expression. This finding supports that epigenetic regulation contributes to increased A4GALT and Gb_3_ levels in vivo. While A4GALT methylation has been noted in other contexts [66], our study demonstrates its functional and tumor-specific relevance in gastrointestinal cancers, linking epigenetic regulation of A4GALT to altered Gb_3_ content.

In CRISPR/Cas9-engineered A4GALT-deficient HCT116 cells, we observed a complete loss of Gb_3_, directly demonstrated by mass spectrometry, and resistance against STx1a. Wildtype HCT116 cells showed significantly higher migratory and invasive capacities compared to A4GALT-deficient clones. These results align with previous reports in colorectal and pancreatic cancer models [24, 67]. Given that A4GALT catalyzes the first step of the globoseries branch of glycosphingolipid biosynthesis, its loss might be expected to lead to accumulation of the shared precursor lactosylceramide and increased synthesis of ganglio- and (neo)lacto-series glycosphingolipids via enzymes such as ST3GAL5. However, in HCT116 cells, we did not observe major alterations of precursor lipids in A4GALT-deficient cells, as compared to the parental cell line. However, such metabolic rewiring could occur in other cell types, and would be likely to alter the composition and organization of lipid rafts and thereby modulate pathways that control cell migration and invasion. Moreover, perturbations of sphingolipid metabolism including reduced conversion of ceramide into complex glycosphingolipids have been linked to endoplasmic reticulum stress, which may provide additional compensatory mechanisms that buffer or reshape the phenotypic consequences of A4GALT loss in HCT116 cells.

Patient-derived transcriptomic data confirmed the pro-invasive role of A4GALT observed in vitro: High A4GALT expression was tightly linked to Hallmarks of EMT, angiogenesis, WNT, and TGF-β programs, emphasizing its role in promoting tumor cell plasticity and microenvironmental interactions. Multiple tumor-promoting genes, including ANAXAL1 (ANAXA8-family), CCKBR, TMPRSS11E and SETBP1 were strongly upregulated in A4GALT high subsets [68–71]. In contrast, MYC target gene sets were downregulated, consistent with the absence of proliferation differences observed in A4GALT-deficient cells. In line with α-GLA as the metabolic antagonist of A4GALT in Gb_3_ metabolism, the gene expression data also revealed the opposite pattern: high α-GLA expression correlated with elevated MYC target signatures but with reduced EMT-related signatures and lower expression of the same tumor-promoting genes. While EMT signatures were strongly associated with high A4GALT expression in TCGA cohorts in our analysis, the mechanistic link remains indirect and needs to be addressed as an important direction of future research on the putative connection between glycolipid biology and EMT.

To assess whether this molecular antagonism translates into clinical consequences, we next examined patient outcomes. The association between Gb_3_ levels and clinical outcomes in gastrointestinal cancers has been investigated previously, but most studies were limited by small patient cohorts and the technical complexity of direct Gb_3_ detection methods [3–6], resulting in inconsistent findings. In contrast, preclinical data have suggested a role for Gb_3_ in promoting tumor invasiveness and metastatic potential [12, 24]. More recent clinical studies in non-GI tumor types, including head and neck and prostate cancers, have associated high Gb_3_ cell surface expression with advanced tumor stages and poor survival [72, 73]. To overcome these limitations, we used the expression of A4GALT and α-GLA as a surrogate parameter to estimate Gb_3_ levels analogous to our gene expression analysis. This enables large cohort analyses and overcomes technical barriers associated with direct Gb_3_ quantification, while providing clinically relevant findings. As expected from tumor-promoting gene signatures described above, conditions associated with high Gb_3_ content (A4GALT high/ α-GLA low) correlated significantly with poor overall survival in colorectal, gastric, and pancreatic cancers. Intriguingly, this was not the case for EAC, which showed exactly the opposite behaviour with A4GALT low/ α-GLA high favouring poor prognosis. Taken together, this underscores the prognostic relevance of Gb_3_ cell surface expression and its underlying enzymatic balance in these entities.

While this study provides important insights, several limitations should be acknowledged. First, Gb_3_ expression in tissue was assessed indirectly through Cy3-conjugated STxB staining and bulk RNA sequencing. Although the absence of Gb_3_ in A4GALT-deficient HCT116 cells was confirmed using mass spectrometry, additional cell lines could be investigated to validate the correlation between Gb_3_ cell surface expression and enzyme levels. Furthermore, these approaches do not account for intratumoral and stromal heterogeneity or various stromal contributions, such as blood vessels, immune and fibroblastic cells, and may overlook cell-type–specific patterns of Gb_3_ expression. Since scRNA-seq indicated some expression of A4GALT in tumor-associated endothelial cells and fibroblasts, which both are well established to contribute to tumor progression, it is conceivable that Gb_3_ may have additional, non tumor-cell-autonomous and yet unclear functions that might impact on patients’ prognosis and could therefore confound survival analysis. Single-cell transcriptomic approaches or computational deconvolution tools would help clarify cell-type specificity and uncover interactions between tumor and stromal compartments. Moreover, surface-based STxB staining may underestimate intracellular Gb3 pools or fail to detect Gb3 in subcellular compartments that could be therapeutically relevant.

In summary, our study identifies Gb_3_ as a multifunctional and clinically relevant lipid species that exemplifies how altered lipid metabolism contributes to cancer progression. Gb_3_ acts as a mediator of malignancy, promoting invasive behavior. Clinically, Gb_3_ serves as a prognostic marker in gastrointestinal cancers, and its surface accessibility enables targeted therapeutic strategies, including STxB-based delivery approaches. We further show that Gb_3_ levels are regulated through reversible epigenetic repression of A4GALT, providing a mechanistic link between transcriptional control and lipid-driven tumor behavior. Together, these findings highlight the potential of Gb_3_ as a diagnostic and therapeutic entry point into metabolic targeting strategies in cancer, shaped by a finely tuned balance of A4GALT and α-GLA expression.

## Supplementary Information

Supplementary Material 1: Supplementary Fig. 1: Genetic alterations in TCGA patients and A4GALT accessibility in DLD1 and HCT116. Supplementary Fig. 2: Generation of A4GALT deficiency. Supplementary Fig. 3: Direct quantification of Gb3 and other lipid species by MALDI2 mass spectrometry. Supplementary Fig. 4: Detailed Gene expression analysis and signatures. Supplementary Fig. 5: Methylation levels. Supplementary Fig. 6: Patient-derived Organoids. Supplementary Fig. 7: Kaplan-Meier survival analysis details. Supplementary Fig. 8: Disease free survival . Supplementary Fig. 9: Progression free survival. Supplementary Fig. 10: Single cell RNAseq analysis. Supplementary Fig. 11: Survival analysis for esophageal adenocarcinoma (EAC).

## Data Availability

The datasets generated or analyzed during the current study, as well as the R scripts used, are available from the corresponding author upon reasonable request.
