# Supplementary material for: The Gb3-synthase A4GALT is an epigenetically regulated driver of tumor invasiveness in gastrointestinal cancer
Source: BMC Cancer. 2026 Jan 27;26:274. doi: 10.1186/s12885-026-15600-7 (PMC12917973; doi:10.1186/s12885-026-15600-7)
Supplement: Supplementary file 1 — Supplementary Material 1: Supplementary Fig. 1: Genetic alterations in TCGA patients and A4GALT accessibility in DLD1 and HCT116. Supplementary Fig. 2: Generation of A4GALT deficiency. Supplementary Fig. 3: Direct quantification of Gb3 and other lipid species by MALDI2 mass spectrometry. Supplementary Fig. 4: Detailed Gene expression analysis and signatures. Supplementary Fig. 5: Methylation levels. Supplementary Fig. 6: Patient-derived Organoids. Supplementary Fig. 7: Kaplan-Meier survival analysis details. Supplementary Fig. 8: Disease free survival . Supplementary Fig. 9: Progression free survival. Supplementary Fig. 10: Single cell RNAseq analysis. Supplementary Fig. 11: Survival analysis for esophageal adenocarcinoma (EAC). [file 12885_2026_15600_MOESM1_ESM.zip › Supplement 5.pdf]

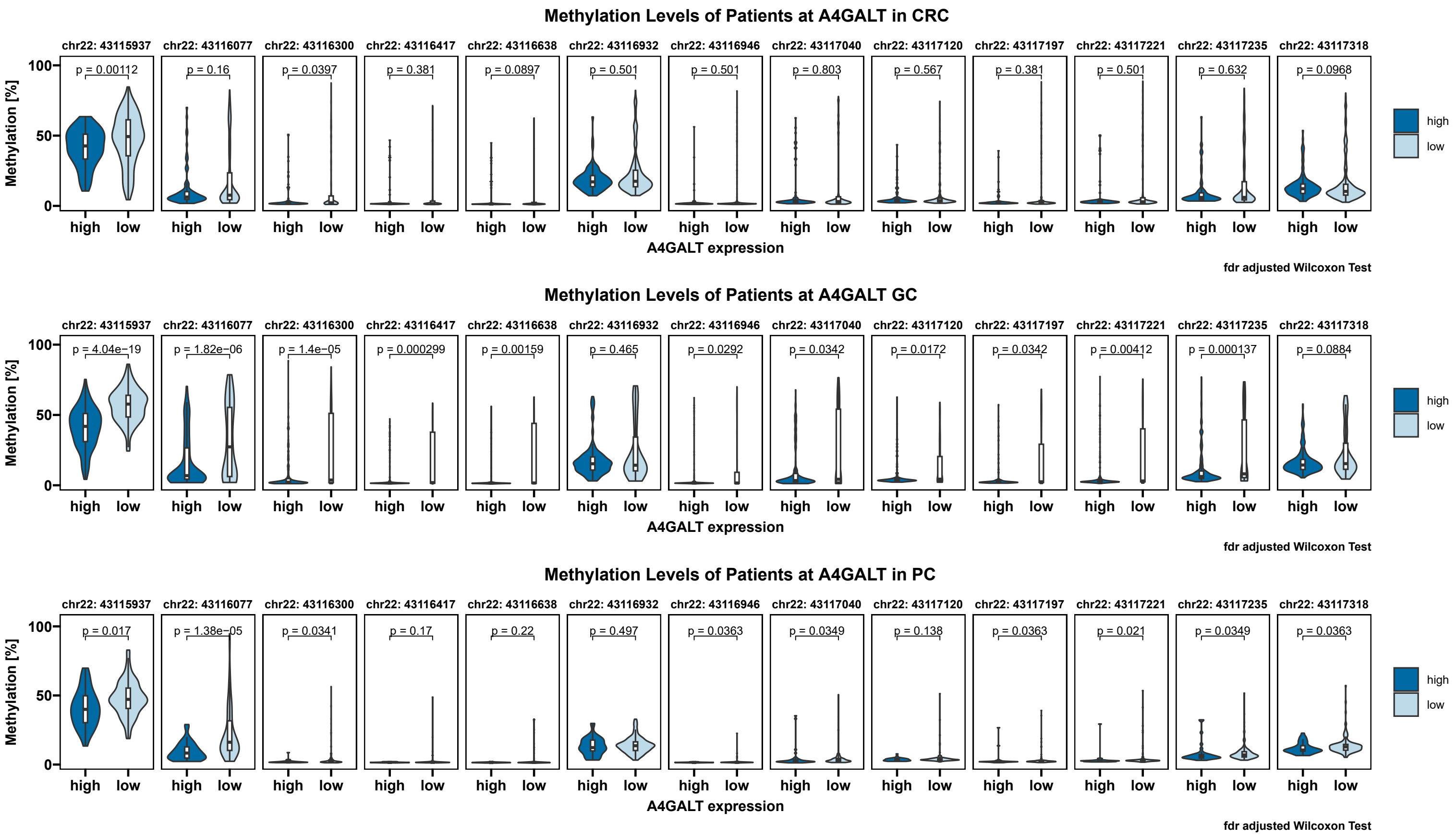

Methylation Levels of Patients at A4GALT in CRC
chr22: 43115937
chr22: 43116077
chr22: 43116300
chr22: 43116417
chr22: 43116638
chr22: 43116932
chr22: 43116946
chr22: 43117040
chr22: 43117120
chr22: 43117197
chr22: 43117221
chr22: 43117235
chr22: 43117318
p = 0.00112
p = 0.16
p = 0.0397
p = 0.381
p = 0.0897
p = 0.501
p = 0.501
p = 0.803
p = 0.567
p = 0.381
p = 0.501
p = 0.632
p = 0.0968
Methylation [%]
100
50
0
high
low
A4GALT expression
fdr adjusted Wilcoxon Test
high
low
Methylation Levels of Patients at A4GALT GC
chr22: 43115937
chr22: 43116077
chr22: 43116300
chr22: 43116417
chr22: 43116638
chr22: 43116932
chr22: 43116946
chr22: 43117040
chr22: 43117120
chr22: 43117197
chr22: 43117221
chr22: 43117235
chr22: 43117318
p = 4.04e−19
p = 1.82e−06
p = 1.4e−05
p = 0.000299
p = 0.00159
p = 0.465
p = 0.0292
p = 0.0342
p = 0.0172
p = 0.0342
p = 0.00412
p = 0.000137
p = 0.0884
Methylation [%]
100
50
0
high
low
A4GALT expression
fdr adjusted Wilcoxon Test
high
low
Methylation Levels of Patients at A4GALT in PC
chr22: 43115937
chr22: 43116077
chr22: 43116300
chr22: 43116417
chr22: 43116638
chr22: 43116932
chr22: 43116946
chr22: 43117040
chr22: 43117120
chr22: 43117197
chr22: 43117221
chr22: 43117235
chr22: 43117318
p = 0.017
p = 1.38e−05
p = 0.0341
p = 0.17
p = 0.22
p = 0.497
p = 0.0363
p = 0.0349
p = 0.138
p = 0.0363
p = 0.021
p = 0.0349
p = 0.0363
Methylation [%]
100
50
0
high
low
A4GALT expression
fdr adjusted Wilcoxon Test
high
low
