# Supplementary material for: The Gb3-synthase A4GALT is an epigenetically regulated driver of tumor invasiveness in gastrointestinal cancer
Source: BMC Cancer. 2026 Jan 27;26:274. doi: 10.1186/s12885-026-15600-7 (PMC12917973; doi:10.1186/s12885-026-15600-7)
Supplement: Supplementary file 1 — Supplementary Material 1: Supplementary Fig. 1: Genetic alterations in TCGA patients and A4GALT accessibility in DLD1 and HCT116. Supplementary Fig. 2: Generation of A4GALT deficiency. Supplementary Fig. 3: Direct quantification of Gb3 and other lipid species by MALDI2 mass spectrometry. Supplementary Fig. 4: Detailed Gene expression analysis and signatures. Supplementary Fig. 5: Methylation levels. Supplementary Fig. 6: Patient-derived Organoids. Supplementary Fig. 7: Kaplan-Meier survival analysis details. Supplementary Fig. 8: Disease free survival . Supplementary Fig. 9: Progression free survival. Supplementary Fig. 10: Single cell RNAseq analysis. Supplementary Fig. 11: Survival analysis for esophageal adenocarcinoma (EAC). [file 12885_2026_15600_MOESM1_ESM.zip › Supplement 7.pdf]

A Baseline characteristics of the TCGA patient cohorts

	CRC (n = 597)	GC (n = 354)	PC (n = 176)	EAC (n = 86)
Sex (n; female:male)	275 (46.06%): 322 (53.94%)	125 (35.31%): 229 (64.69%)	80 (45.45%): 96 (54.55%)	12 (13.95 %): 74 (86.05 %)
Age at diagnosis, mean (Q1:Q3)	66 (58:76)	66 (58:73)	65 (57:74)	67 (58:77)
Most frequent stage (AJCC)	stage II (36.18%)	stage III (41.24%)	stage II (82.95%)	(missing)

	Group size (n)	Maximum follow-up (years)	Median survival (years)	5-year survival (%)	Cut-off A4GALT, FPKM	Cut-off α-GLA, FPKM
CRC	597	12.3	6.94	62	4.82	12.81
GC	354	10.2	2.41	38	2.99	13.82
PC	176	7.5	1.66	28	14.54	11.02
EAC	121	10.2	1.79	20	8.36	3.11

B Kaplan–Meier survival analysis of A4GALT and α-GLA only

		group size (n)	Number of deaths	Number of censored	Median survival (years)	Hazard Ratio high:low (95% CI)
CRC	A4GALT high	120	32	88	5.61	1.71 (1.08 to 2.70)
	A4GALT low	477	92	385	6.94	
GC	A4GALT high	242	104	138	2.18	1.26 (0.89 to 1.77)
	A4GALT low	112	42	70	3.54	
PC	A4GALT high	37	23	14	1.18	2.32 (1.29 to 4.16)
	A4GALT low	139	69	70	1.81	
EAC	A4GALT high	11	2	9	5.85	0.40 (0.18 to 0.89)
	A4GALT low	75	42	33	1.67	

		group size (n)	Number of deaths	Number of censored	Median survival (years)	Hazard Ratio high:low (95% CI)
CRC	α-GLA high	446	82	364	/	0.60 (0.40 to 0.91)
	α-GLA low	151	42	109	5.15	
GC	α-GLA high	125	39	86	3.85	0.57 (0.41 to 0.79)
	α-GLA low	229	107	122	2.09	
PC	α-GLA high	62	30	32	1.90	0.64 (0.42 to 0.98)
	α-GLA low	114	62	52	1.62	
EAC	α-GLA high	60	38	22	1.36	3.22 (1.72 to 6.03)
	α-GLA low	26	6	20	4.88	

C

Kaplan–Meier survival analysis of A4GALT and α-GLA combination

		group size (n)	Number of deaths	Number of censored	Median survival (years)
CRC	A4GALT-/α-GLA-	122	31	91	5.15
	A4GALT+/α-GLA-	29	11	18	5.61
	A4GALT-/α-GLA+	355	61	294	/
	A4GALT+/α-GLA+	91	21	70	8.33
GC	A4GALT-/α-GLA-	72	30	42	2.86
	A4GALT+/α-GLA-	157	77	80	1.74
	A4GALT-/α-GLA+	40	12	28	/
	A4GALT+/α-GLA+	85	27	58	/
PC	A4GALT-/α-GLA-	90	46	44	1.63
	A4GALT+/α-GLA-	24	16	8	1.18
	A4GALT-/α-α-GLA+	49	23	26	2.90
	A4GALT+/α-GLA+	13	7	6	1.08
EAC	A4GALT-/α-GLA-	20	6	14	4.88
	A4GALT+/α-GLA-	6	0	6	/
	A4GALT-/α-GLA+	55	36	19	1.35
	A4GALT+/α-GLA+	5	2	3	5.85

CRC: A4GALT+/α-GLA- vs.	HR	95% CI	p-value
all	2.455	1.32-4.57	0.0046
A4GALT+ only	0.516	0.248-1.07	0.0761
α-GLA- only	0.519	0.261-1.03	0.0622

GC: A4GALT+/α-GLA- vs.	HR	95% CI	p-value
all	1.78	1.28-2.246	0.00058
A4GALT+ only	0.491	0.317-0.764	0.0016
α-GLA- only	0.714	0.467-1.09	0.12

PC: A4GALT+/α-GLA- vs.	HR	95% CI	p-value
all	1.869	1.09-3.22	0.024
A4GALT+ only	1.013	0.416-2.47	0.98
α-GLA- only	0.619	0.350-1.10	0.1

EAC: A4GALT+/α-GLA- vs.	HR	95% CI	p-value
all	/	/	0.996
A4GALT+ only	/	/	0.997
α-GLA- only	/	/	0.997
