# Supplementary figures and images for: The Gb3-synthase A4GALT is an epigenetically regulated driver of tumor invasiveness in gastrointestinal cancer
Source: BMC Cancer. 2026 Jan 27;26:274. doi: 10.1186/s12885-026-15600-7 (PMC12917973; doi:10.1186/s12885-026-15600-7)

# Enrichment of Hallmarks in CRC of A4GALT+ Patients

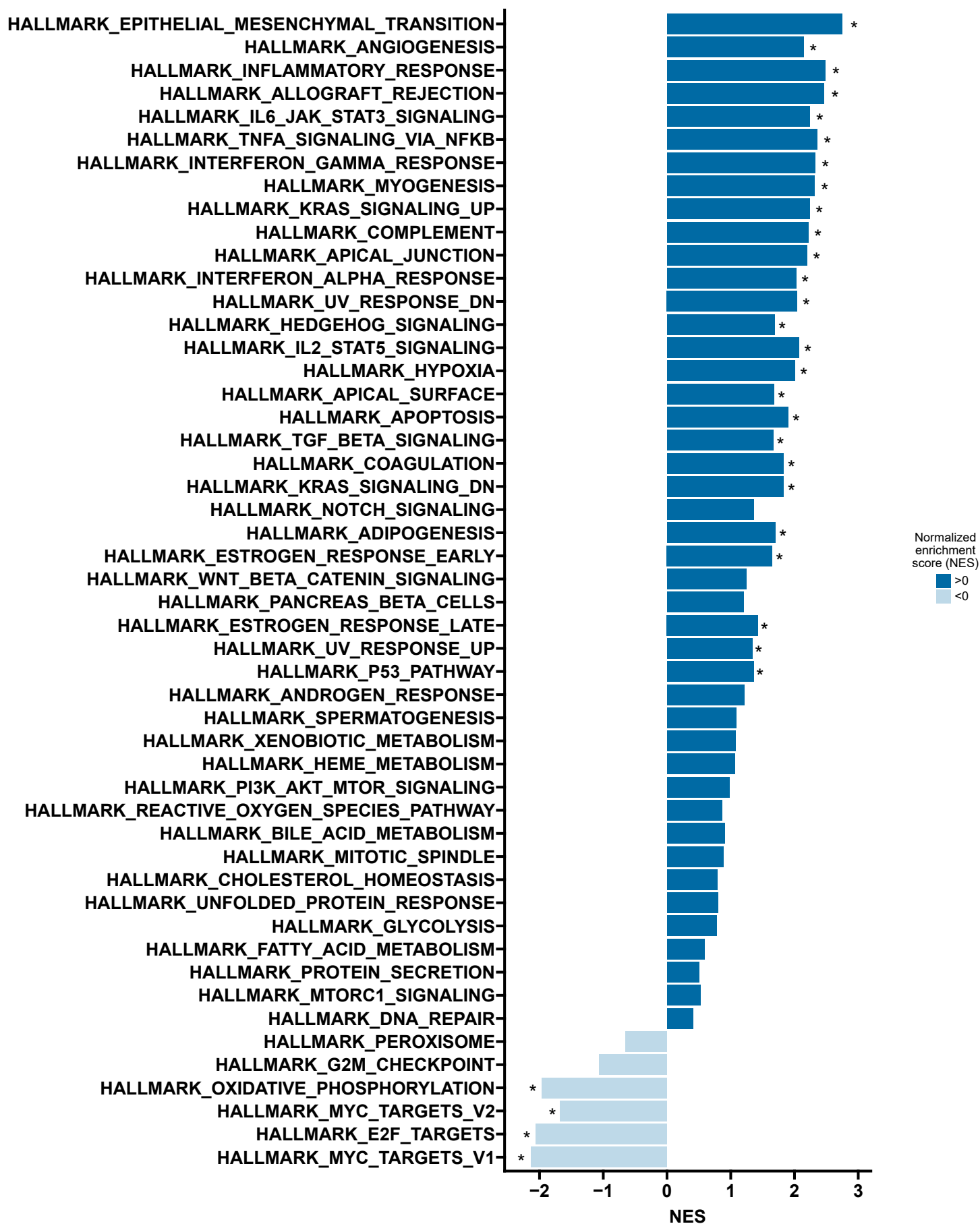

Supplement: Supplementary file 1 — Supplementary Material 1: Supplementary Fig. 1: Genetic alterations in TCGA patients and A4GALT accessibility in DLD1 and HCT116. Supplementary Fig. 2: Generation of A4GALT deficiency. Supplementary Fig. 3: Direct quantification of Gb3 and other lipid species by MALDI2 mass spectrometry. Supplementary Fig. 4: Detailed Gene expression analysis and signatures. Supplementary Fig. 5: Methylation levels. Supplementary Fig. 6: Patient-derived Organoids. Supplementary Fig. 7: Kaplan-Meier survival analysis details. Supplementary Fig. 8: Disease free survival . Supplementary Fig. 9: Progression free survival. Supplementary Fig. 10: Single cell RNAseq analysis. Supplementary Fig. 11: Survival analysis for esophageal adenocarcinoma (EAC). [file 12885_2026_15600_MOESM1_ESM.zip › Suppl 4 Patients_CRC_Hallmarks_A4GALT.pdf]

# Enrichment of Hallmarks in CRC of aGLA+ Patients

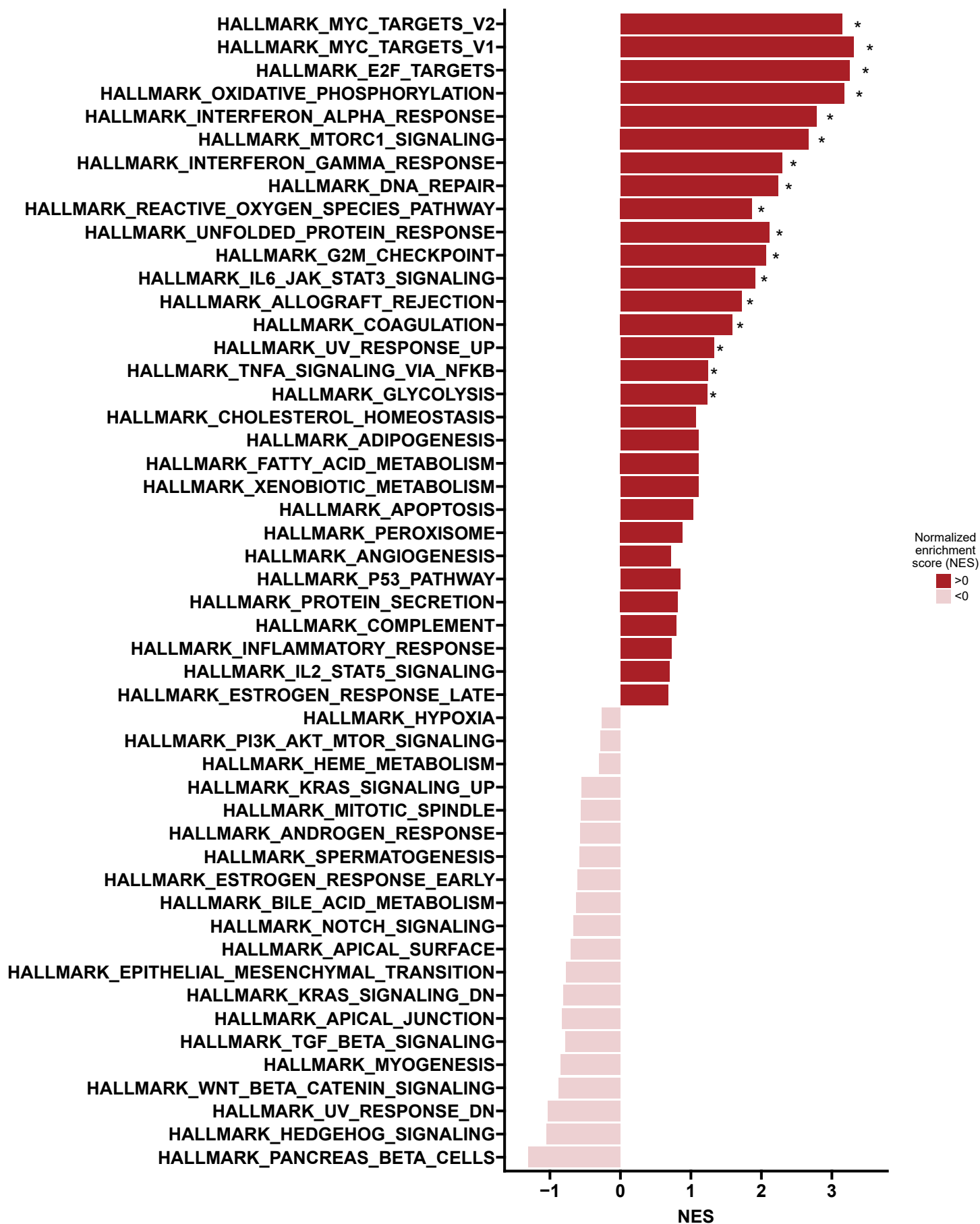

Supplement: Supplementary file 1 — Supplementary Material 1: Supplementary Fig. 1: Genetic alterations in TCGA patients and A4GALT accessibility in DLD1 and HCT116. Supplementary Fig. 2: Generation of A4GALT deficiency. Supplementary Fig. 3: Direct quantification of Gb3 and other lipid species by MALDI2 mass spectrometry. Supplementary Fig. 4: Detailed Gene expression analysis and signatures. Supplementary Fig. 5: Methylation levels. Supplementary Fig. 6: Patient-derived Organoids. Supplementary Fig. 7: Kaplan-Meier survival analysis details. Supplementary Fig. 8: Disease free survival . Supplementary Fig. 9: Progression free survival. Supplementary Fig. 10: Single cell RNAseq analysis. Supplementary Fig. 11: Survival analysis for esophageal adenocarcinoma (EAC). [file 12885_2026_15600_MOESM1_ESM.zip › Suppl 4 Patients_CRC_Hallmarks_aGLA.pdf]

# A4GALT high vs low

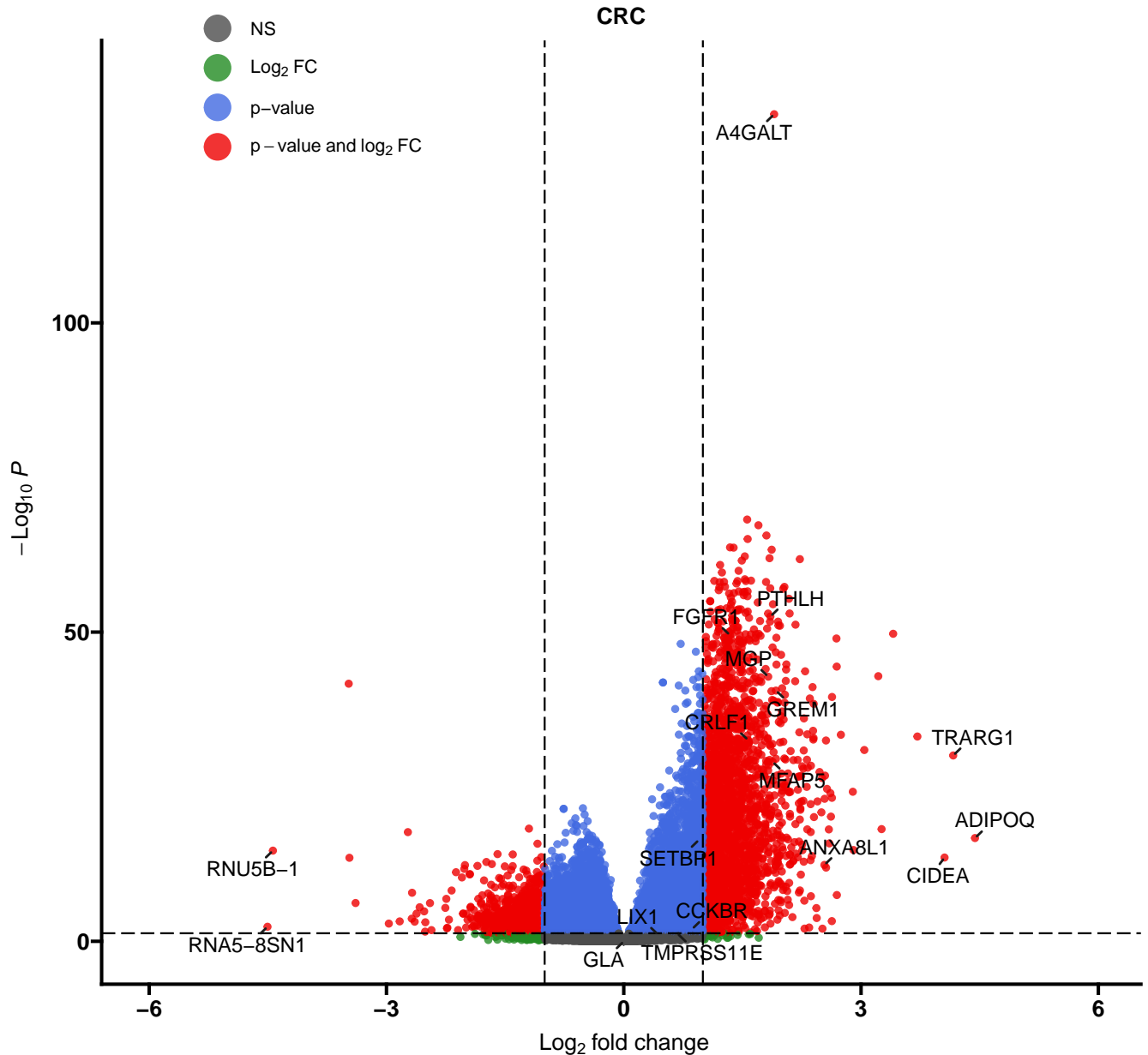

total = 36608 variables

Supplement: Supplementary file 1 — Supplementary Material 1: Supplementary Fig. 1: Genetic alterations in TCGA patients and A4GALT accessibility in DLD1 and HCT116. Supplementary Fig. 2: Generation of A4GALT deficiency. Supplementary Fig. 3: Direct quantification of Gb3 and other lipid species by MALDI2 mass spectrometry. Supplementary Fig. 4: Detailed Gene expression analysis and signatures. Supplementary Fig. 5: Methylation levels. Supplementary Fig. 6: Patient-derived Organoids. Supplementary Fig. 7: Kaplan-Meier survival analysis details. Supplementary Fig. 8: Disease free survival . Supplementary Fig. 9: Progression free survival. Supplementary Fig. 10: Single cell RNAseq analysis. Supplementary Fig. 11: Survival analysis for esophageal adenocarcinoma (EAC). [file 12885_2026_15600_MOESM1_ESM.zip › Suppl 4 Patients_CRC_Vulcano_A4GALT.pdf]

# aGLA high vs low

CRC

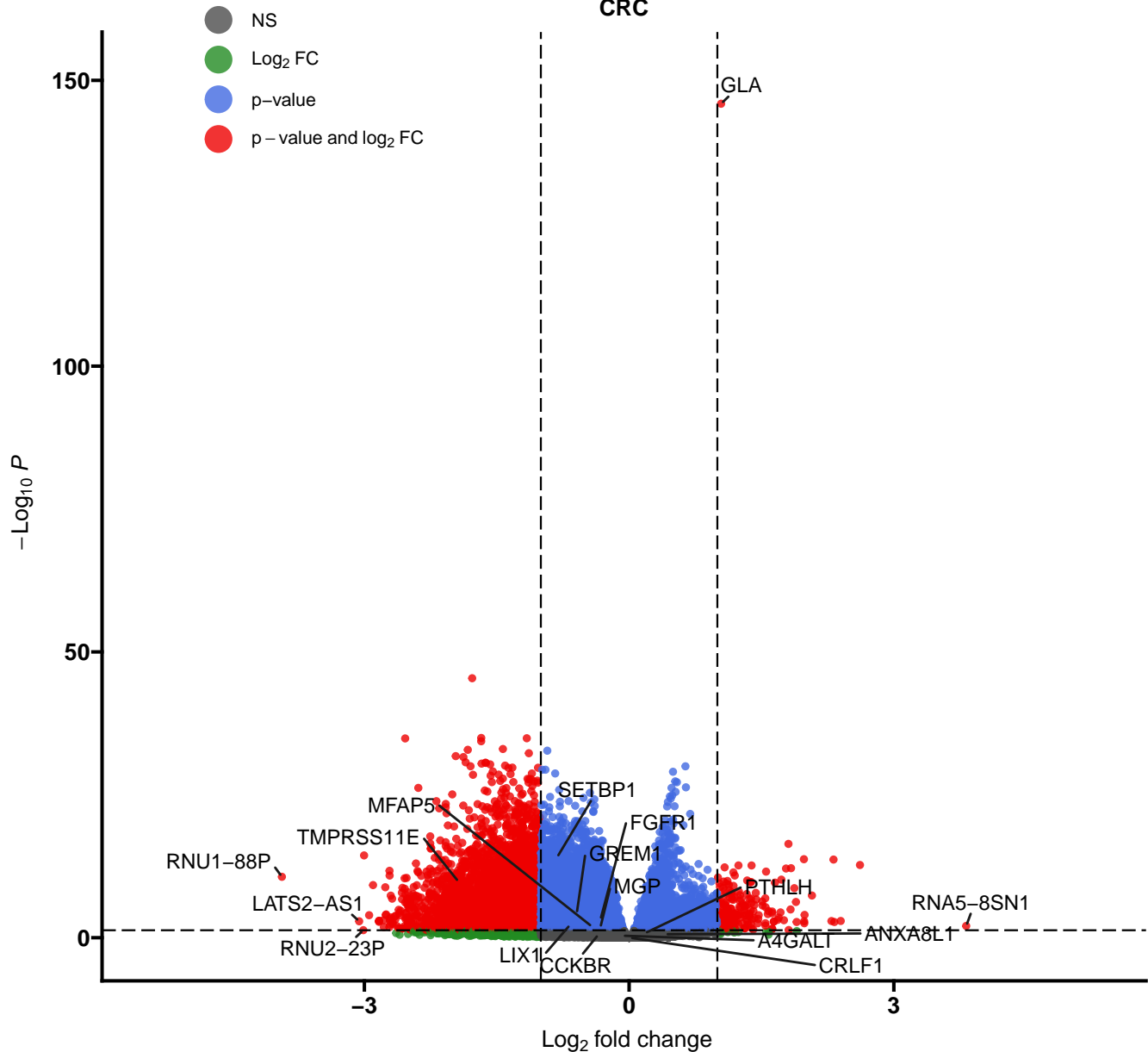

total = 36608 variables

Supplement: Supplementary file 1 — Supplementary Material 1: Supplementary Fig. 1: Genetic alterations in TCGA patients and A4GALT accessibility in DLD1 and HCT116. Supplementary Fig. 2: Generation of A4GALT deficiency. Supplementary Fig. 3: Direct quantification of Gb3 and other lipid species by MALDI2 mass spectrometry. Supplementary Fig. 4: Detailed Gene expression analysis and signatures. Supplementary Fig. 5: Methylation levels. Supplementary Fig. 6: Patient-derived Organoids. Supplementary Fig. 7: Kaplan-Meier survival analysis details. Supplementary Fig. 8: Disease free survival . Supplementary Fig. 9: Progression free survival. Supplementary Fig. 10: Single cell RNAseq analysis. Supplementary Fig. 11: Survival analysis for esophageal adenocarcinoma (EAC). [file 12885_2026_15600_MOESM1_ESM.zip › Suppl 4 Patients_CRC_Vulcano_aGLA.pdf]

# Enrichment of Hallmarks in GC of A4GALT+ Patients

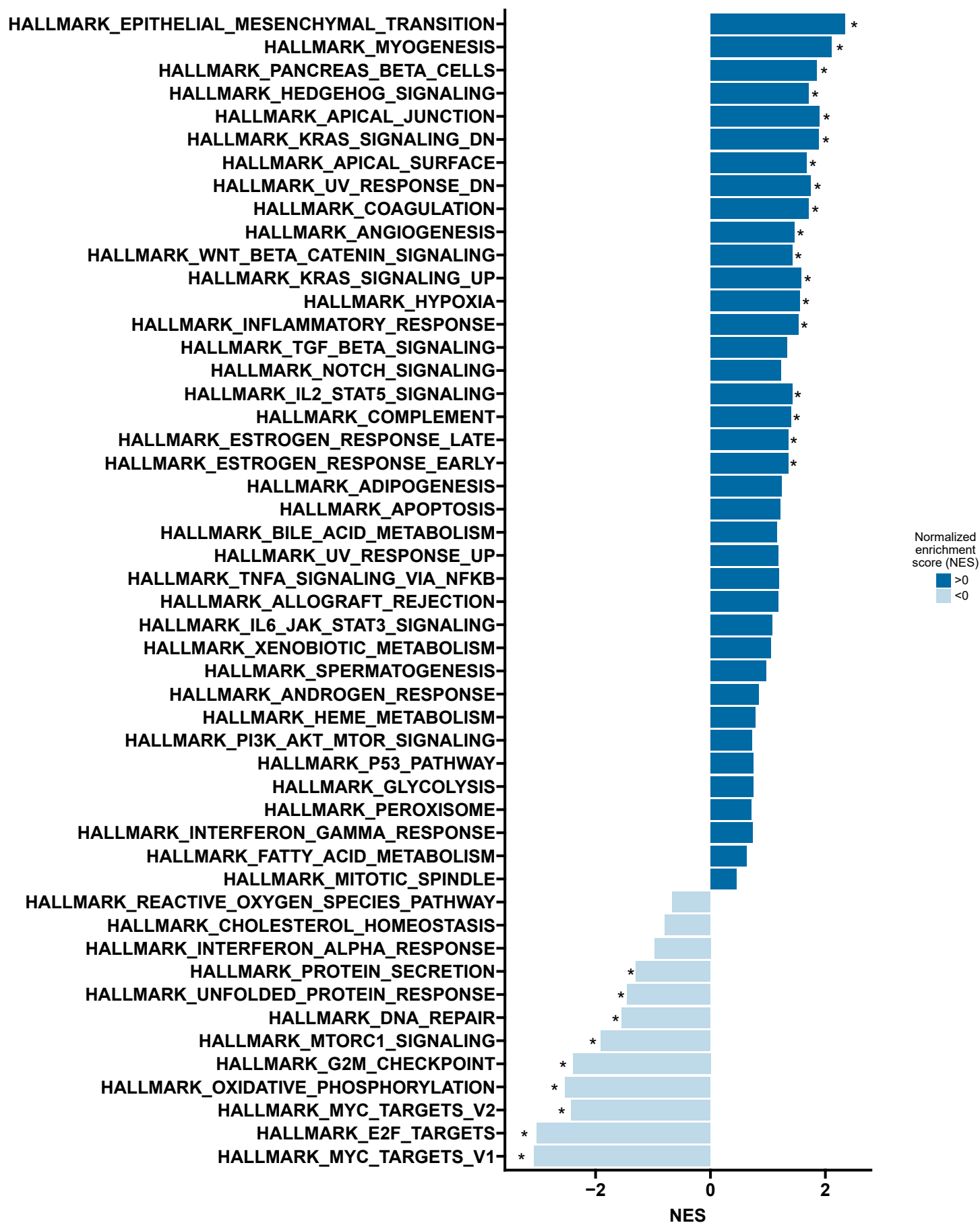

Supplement: Supplementary file 1 — Supplementary Material 1: Supplementary Fig. 1: Genetic alterations in TCGA patients and A4GALT accessibility in DLD1 and HCT116. Supplementary Fig. 2: Generation of A4GALT deficiency. Supplementary Fig. 3: Direct quantification of Gb3 and other lipid species by MALDI2 mass spectrometry. Supplementary Fig. 4: Detailed Gene expression analysis and signatures. Supplementary Fig. 5: Methylation levels. Supplementary Fig. 6: Patient-derived Organoids. Supplementary Fig. 7: Kaplan-Meier survival analysis details. Supplementary Fig. 8: Disease free survival . Supplementary Fig. 9: Progression free survival. Supplementary Fig. 10: Single cell RNAseq analysis. Supplementary Fig. 11: Survival analysis for esophageal adenocarcinoma (EAC). [file 12885_2026_15600_MOESM1_ESM.zip › Suppl 4 Patients_GC_Hallmarks_A4GALT.pdf]

# Enrichment of Hallmarks in GC of aGLA+ Patients

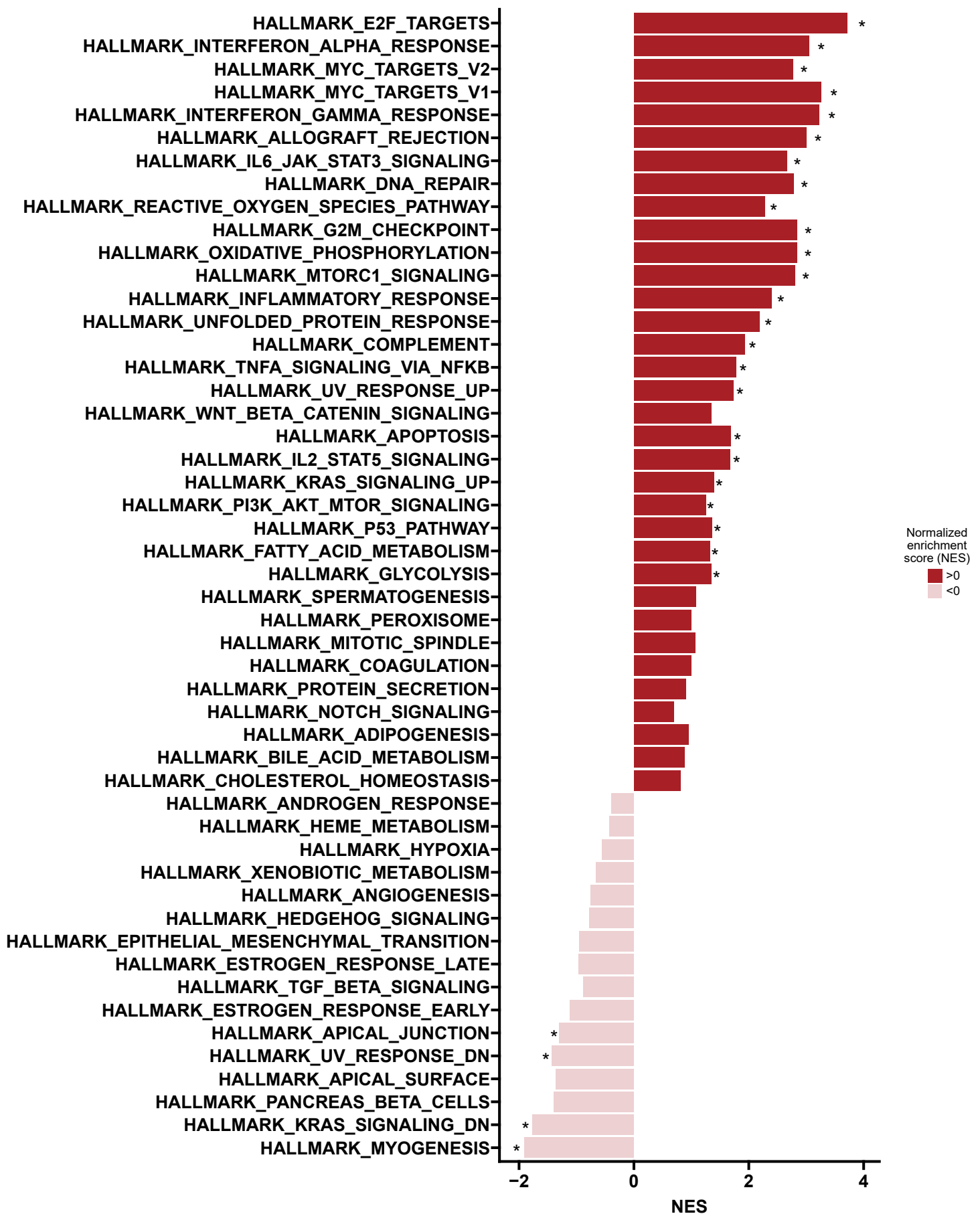

Supplement: Supplementary file 1 — Supplementary Material 1: Supplementary Fig. 1: Genetic alterations in TCGA patients and A4GALT accessibility in DLD1 and HCT116. Supplementary Fig. 2: Generation of A4GALT deficiency. Supplementary Fig. 3: Direct quantification of Gb3 and other lipid species by MALDI2 mass spectrometry. Supplementary Fig. 4: Detailed Gene expression analysis and signatures. Supplementary Fig. 5: Methylation levels. Supplementary Fig. 6: Patient-derived Organoids. Supplementary Fig. 7: Kaplan-Meier survival analysis details. Supplementary Fig. 8: Disease free survival . Supplementary Fig. 9: Progression free survival. Supplementary Fig. 10: Single cell RNAseq analysis. Supplementary Fig. 11: Survival analysis for esophageal adenocarcinoma (EAC). [file 12885_2026_15600_MOESM1_ESM.zip › Suppl 4 Patients_GC_Hallmarks_aGLA.pdf]

# A4GALT high vs low

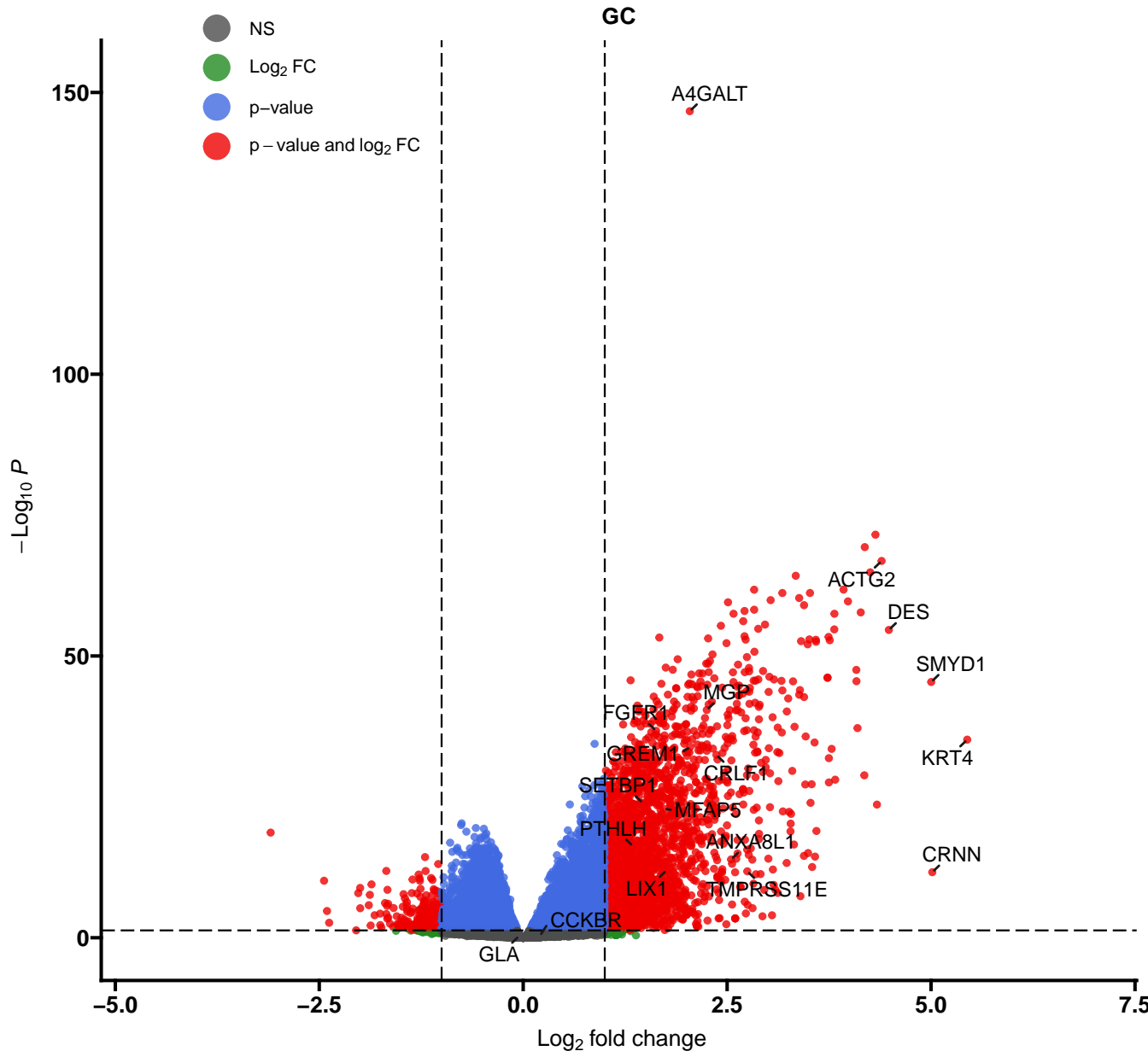

total = 46503 variables

Supplement: Supplementary file 1 — Supplementary Material 1: Supplementary Fig. 1: Genetic alterations in TCGA patients and A4GALT accessibility in DLD1 and HCT116. Supplementary Fig. 2: Generation of A4GALT deficiency. Supplementary Fig. 3: Direct quantification of Gb3 and other lipid species by MALDI2 mass spectrometry. Supplementary Fig. 4: Detailed Gene expression analysis and signatures. Supplementary Fig. 5: Methylation levels. Supplementary Fig. 6: Patient-derived Organoids. Supplementary Fig. 7: Kaplan-Meier survival analysis details. Supplementary Fig. 8: Disease free survival . Supplementary Fig. 9: Progression free survival. Supplementary Fig. 10: Single cell RNAseq analysis. Supplementary Fig. 11: Survival analysis for esophageal adenocarcinoma (EAC). [file 12885_2026_15600_MOESM1_ESM.zip › Suppl 4 Patients_GC_Vulcano_A4GALT.pdf]

# aGLA high vs low

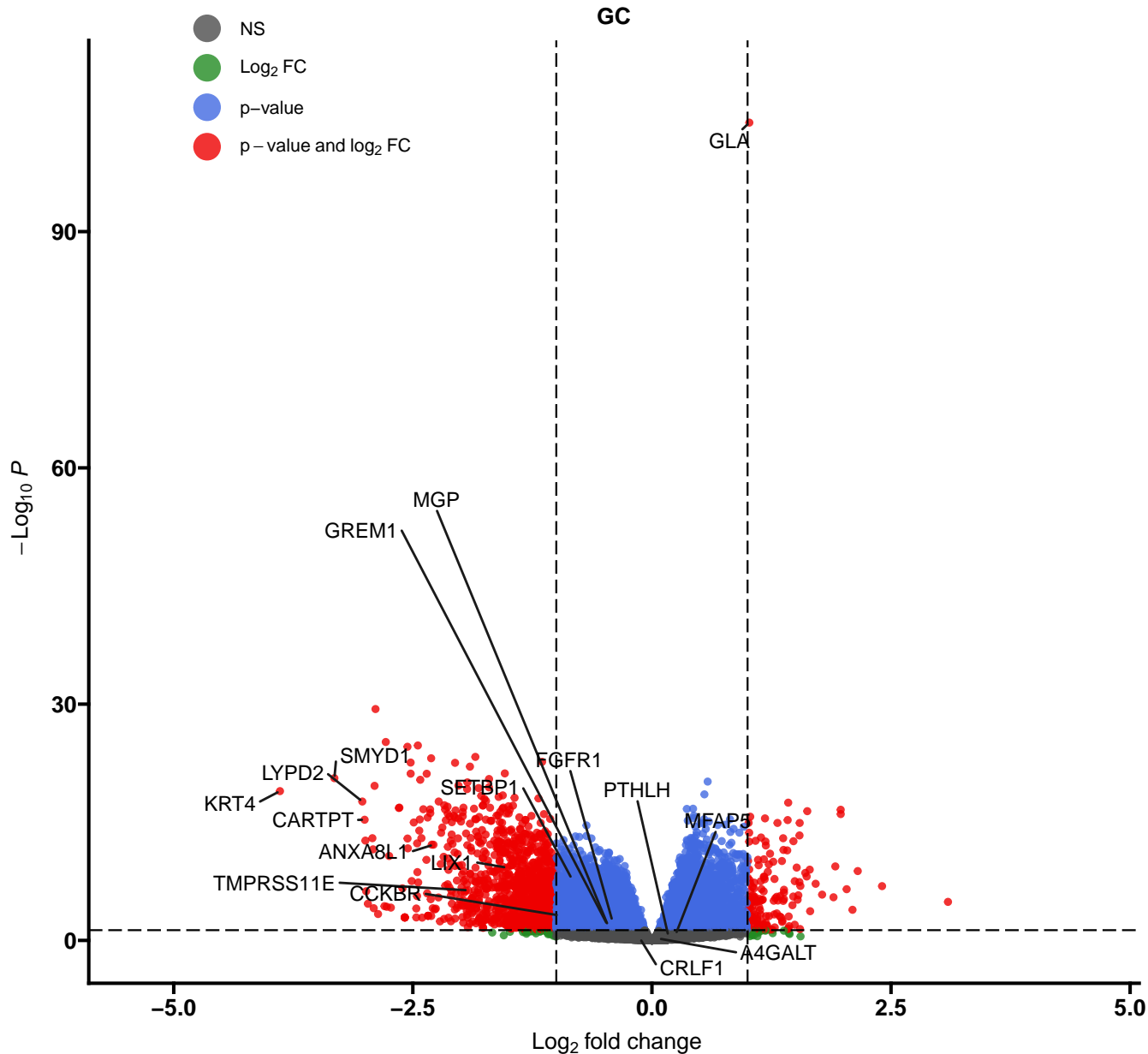

total = 46503 variables

Supplement: Supplementary file 1 — Supplementary Material 1: Supplementary Fig. 1: Genetic alterations in TCGA patients and A4GALT accessibility in DLD1 and HCT116. Supplementary Fig. 2: Generation of A4GALT deficiency. Supplementary Fig. 3: Direct quantification of Gb3 and other lipid species by MALDI2 mass spectrometry. Supplementary Fig. 4: Detailed Gene expression analysis and signatures. Supplementary Fig. 5: Methylation levels. Supplementary Fig. 6: Patient-derived Organoids. Supplementary Fig. 7: Kaplan-Meier survival analysis details. Supplementary Fig. 8: Disease free survival . Supplementary Fig. 9: Progression free survival. Supplementary Fig. 10: Single cell RNAseq analysis. Supplementary Fig. 11: Survival analysis for esophageal adenocarcinoma (EAC). [file 12885_2026_15600_MOESM1_ESM.zip › Suppl 4 Patients_GC_Vulcano_aGLA.pdf]

# Enrichment of Hallmarks in PC of A4GALT+ Patients

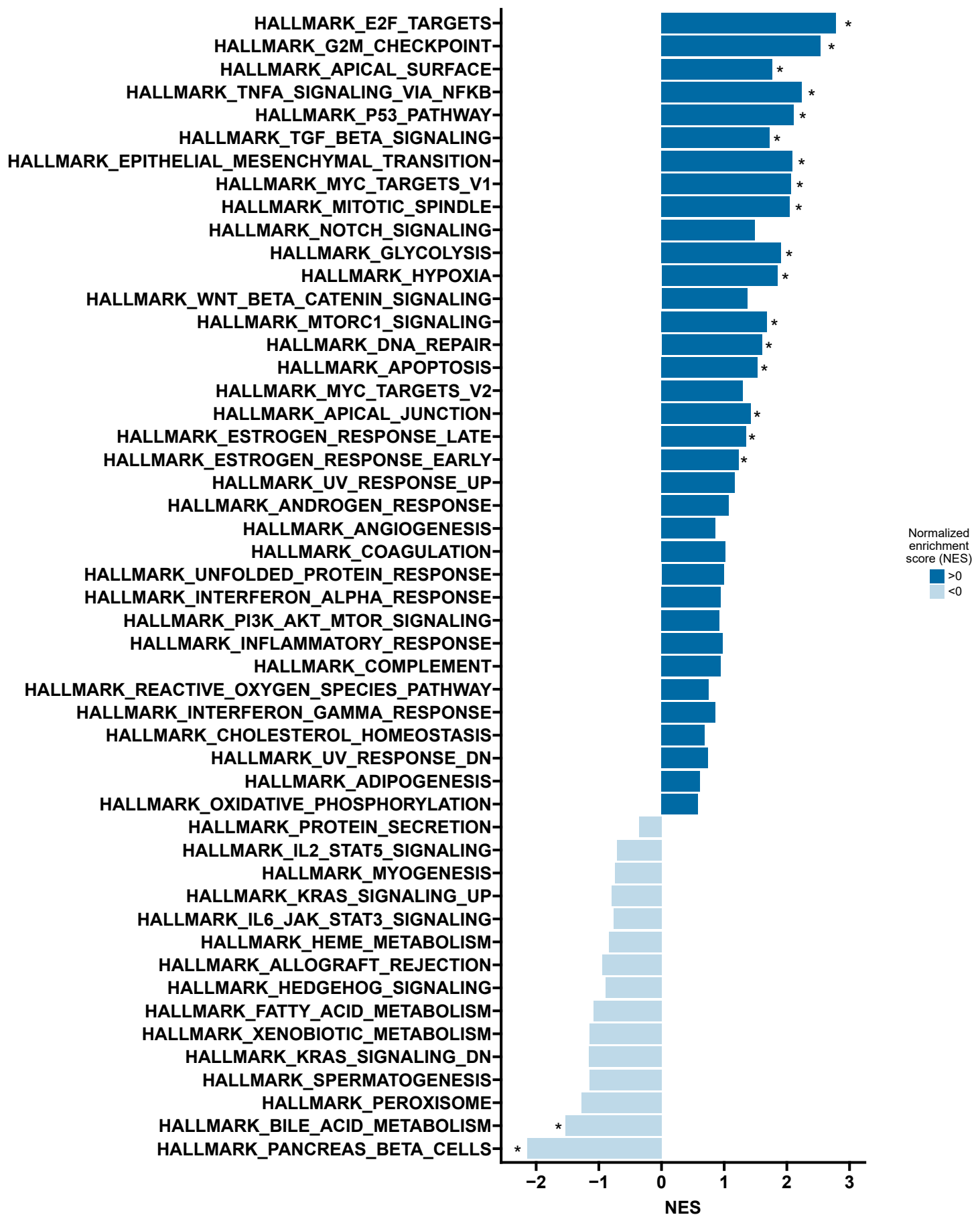

Supplement: Supplementary file 1 — Supplementary Material 1: Supplementary Fig. 1: Genetic alterations in TCGA patients and A4GALT accessibility in DLD1 and HCT116. Supplementary Fig. 2: Generation of A4GALT deficiency. Supplementary Fig. 3: Direct quantification of Gb3 and other lipid species by MALDI2 mass spectrometry. Supplementary Fig. 4: Detailed Gene expression analysis and signatures. Supplementary Fig. 5: Methylation levels. Supplementary Fig. 6: Patient-derived Organoids. Supplementary Fig. 7: Kaplan-Meier survival analysis details. Supplementary Fig. 8: Disease free survival . Supplementary Fig. 9: Progression free survival. Supplementary Fig. 10: Single cell RNAseq analysis. Supplementary Fig. 11: Survival analysis for esophageal adenocarcinoma (EAC). [file 12885_2026_15600_MOESM1_ESM.zip › Suppl 4 Patients_PC_Hallmarks_A4GALT.pdf]

# Enrichment of Hallmarks in PC of aGLA+ Patients

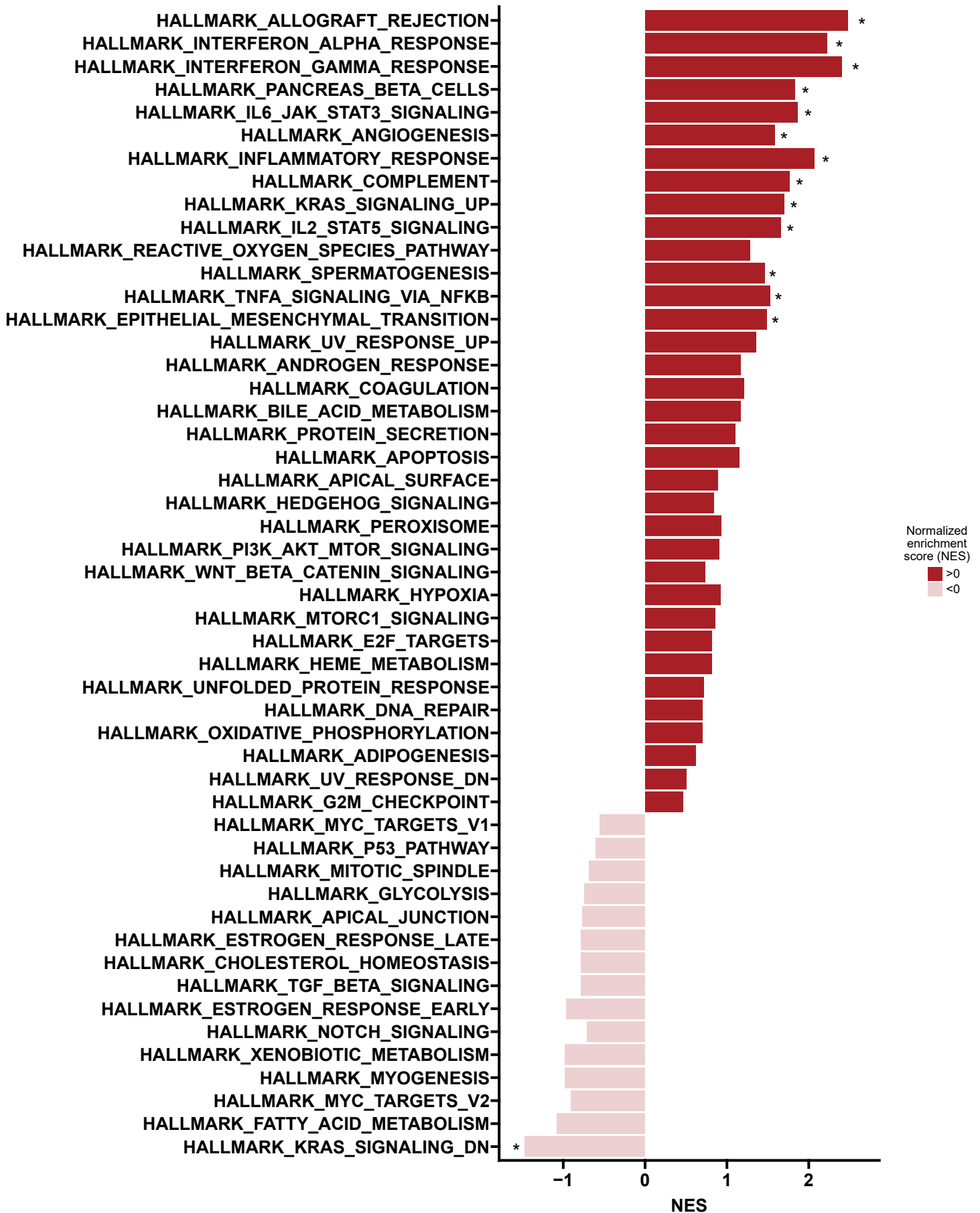

Supplement: Supplementary file 1 — Supplementary Material 1: Supplementary Fig. 1: Genetic alterations in TCGA patients and A4GALT accessibility in DLD1 and HCT116. Supplementary Fig. 2: Generation of A4GALT deficiency. Supplementary Fig. 3: Direct quantification of Gb3 and other lipid species by MALDI2 mass spectrometry. Supplementary Fig. 4: Detailed Gene expression analysis and signatures. Supplementary Fig. 5: Methylation levels. Supplementary Fig. 6: Patient-derived Organoids. Supplementary Fig. 7: Kaplan-Meier survival analysis details. Supplementary Fig. 8: Disease free survival . Supplementary Fig. 9: Progression free survival. Supplementary Fig. 10: Single cell RNAseq analysis. Supplementary Fig. 11: Survival analysis for esophageal adenocarcinoma (EAC). [file 12885_2026_15600_MOESM1_ESM.zip › Suppl 4 Patients_PC_Hallmarks_aGLA.pdf]

# A4GALT high vs low

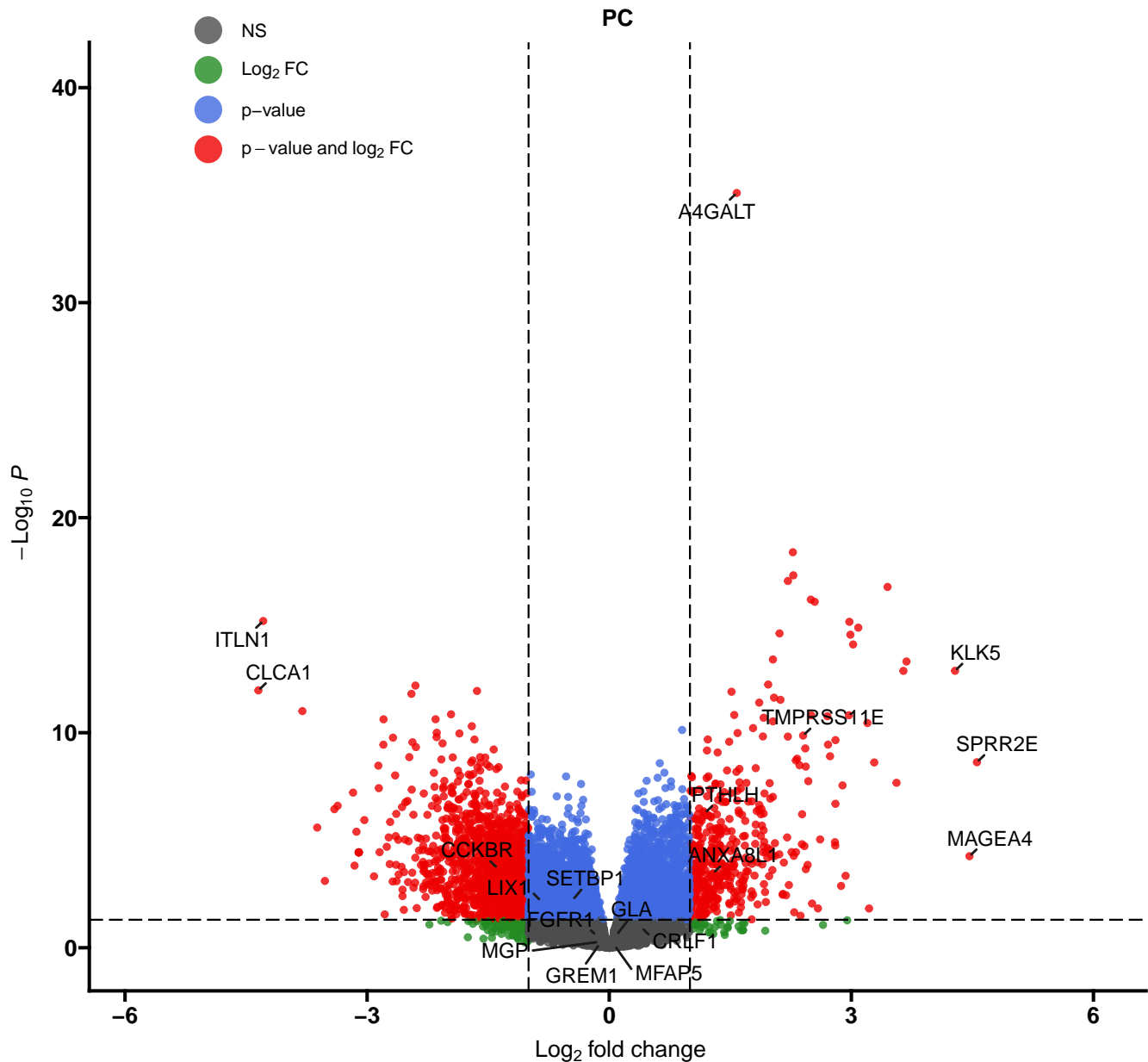

total = 30124 variables

Supplement: Supplementary file 1 — Supplementary Material 1: Supplementary Fig. 1: Genetic alterations in TCGA patients and A4GALT accessibility in DLD1 and HCT116. Supplementary Fig. 2: Generation of A4GALT deficiency. Supplementary Fig. 3: Direct quantification of Gb3 and other lipid species by MALDI2 mass spectrometry. Supplementary Fig. 4: Detailed Gene expression analysis and signatures. Supplementary Fig. 5: Methylation levels. Supplementary Fig. 6: Patient-derived Organoids. Supplementary Fig. 7: Kaplan-Meier survival analysis details. Supplementary Fig. 8: Disease free survival . Supplementary Fig. 9: Progression free survival. Supplementary Fig. 10: Single cell RNAseq analysis. Supplementary Fig. 11: Survival analysis for esophageal adenocarcinoma (EAC). [file 12885_2026_15600_MOESM1_ESM.zip › Suppl 4 Patients_PC_Vulcano_A4GALT.pdf]

# aGLA high vs low

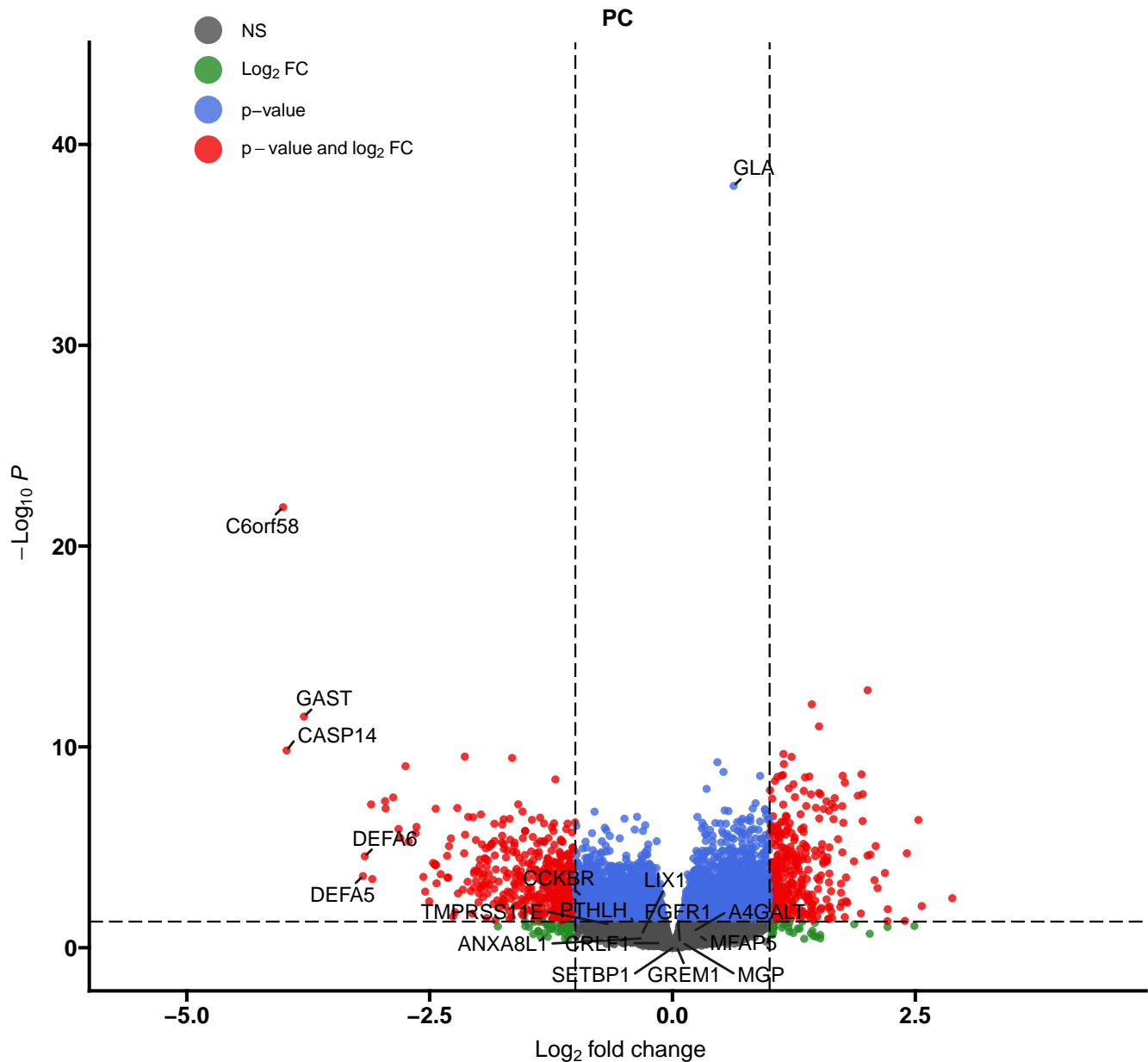

total = 30124 variables

Supplement: Supplementary file 1 — Supplementary Material 1: Supplementary Fig. 1: Genetic alterations in TCGA patients and A4GALT accessibility in DLD1 and HCT116. Supplementary Fig. 2: Generation of A4GALT deficiency. Supplementary Fig. 3: Direct quantification of Gb3 and other lipid species by MALDI2 mass spectrometry. Supplementary Fig. 4: Detailed Gene expression analysis and signatures. Supplementary Fig. 5: Methylation levels. Supplementary Fig. 6: Patient-derived Organoids. Supplementary Fig. 7: Kaplan-Meier survival analysis details. Supplementary Fig. 8: Disease free survival . Supplementary Fig. 9: Progression free survival. Supplementary Fig. 10: Single cell RNAseq analysis. Supplementary Fig. 11: Survival analysis for esophageal adenocarcinoma (EAC). [file 12885_2026_15600_MOESM1_ESM.zip › Suppl 4 Patients_PC_Vulcano_aGLA.pdf]

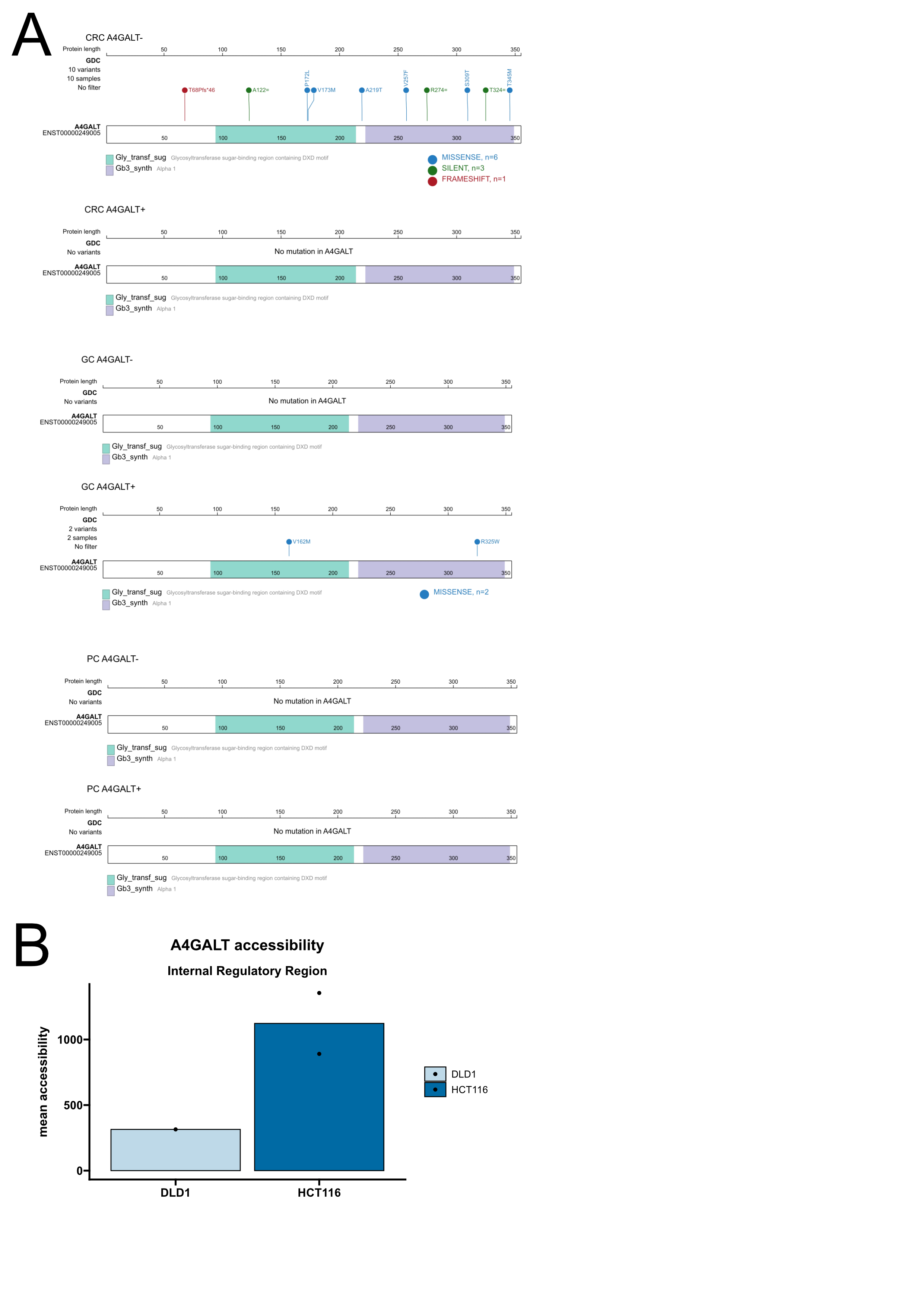

Supplement: Supplementary file 1 — Supplementary Material 1: Supplementary Fig. 1: Genetic alterations in TCGA patients and A4GALT accessibility in DLD1 and HCT116. Supplementary Fig. 2: Generation of A4GALT deficiency. Supplementary Fig. 3: Direct quantification of Gb3 and other lipid species by MALDI2 mass spectrometry. Supplementary Fig. 4: Detailed Gene expression analysis and signatures. Supplementary Fig. 5: Methylation levels. Supplementary Fig. 6: Patient-derived Organoids. Supplementary Fig. 7: Kaplan-Meier survival analysis details. Supplementary Fig. 8: Disease free survival . Supplementary Fig. 9: Progression free survival. Supplementary Fig. 10: Single cell RNAseq analysis. Supplementary Fig. 11: Survival analysis for esophageal adenocarcinoma (EAC). [file 12885_2026_15600_MOESM1_ESM.zip › Supplement 1.tiff]

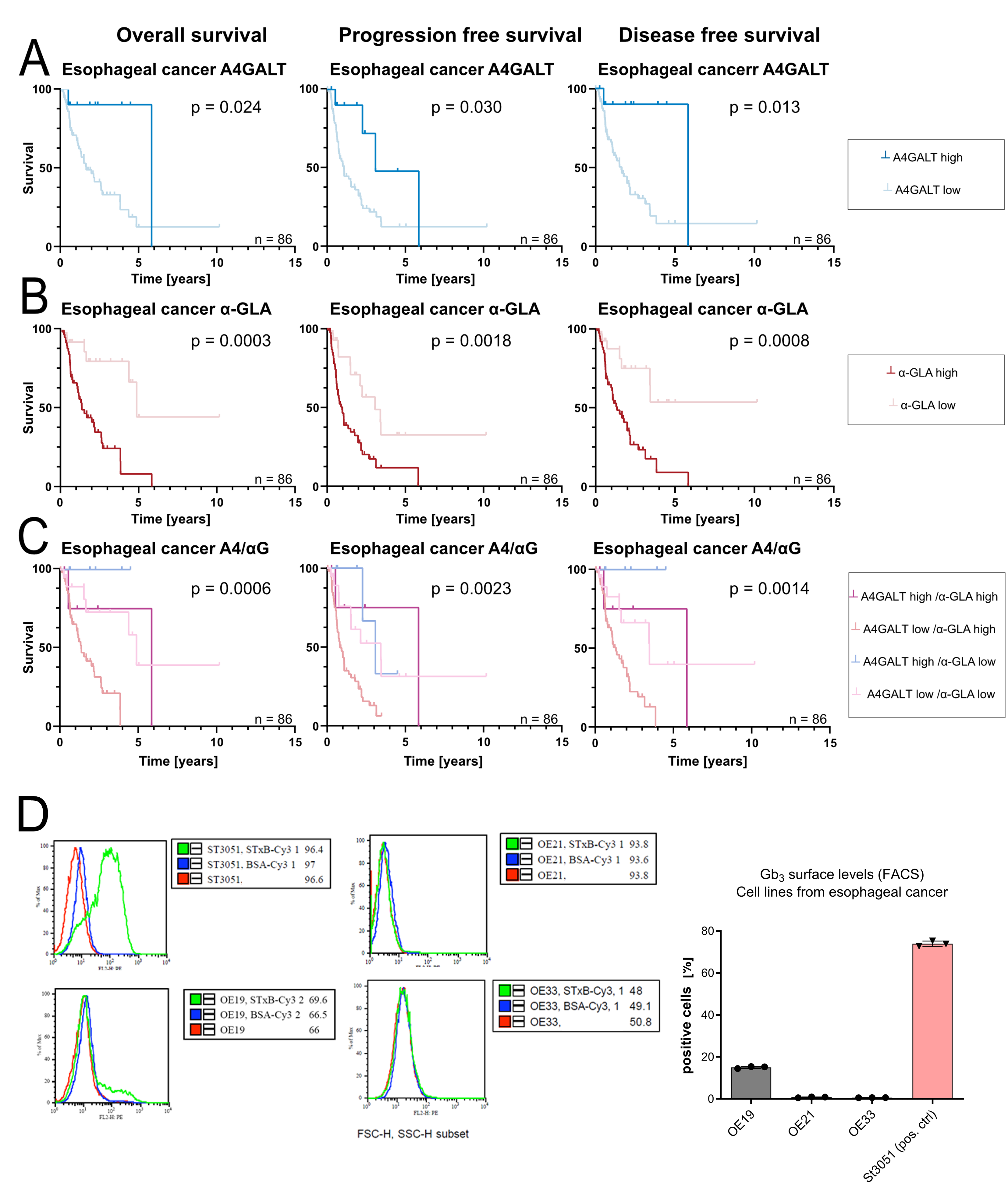

Supplement: Supplementary file 1 — Supplementary Material 1: Supplementary Fig. 1: Genetic alterations in TCGA patients and A4GALT accessibility in DLD1 and HCT116. Supplementary Fig. 2: Generation of A4GALT deficiency. Supplementary Fig. 3: Direct quantification of Gb3 and other lipid species by MALDI2 mass spectrometry. Supplementary Fig. 4: Detailed Gene expression analysis and signatures. Supplementary Fig. 5: Methylation levels. Supplementary Fig. 6: Patient-derived Organoids. Supplementary Fig. 7: Kaplan-Meier survival analysis details. Supplementary Fig. 8: Disease free survival . Supplementary Fig. 9: Progression free survival. Supplementary Fig. 10: Single cell RNAseq analysis. Supplementary Fig. 11: Survival analysis for esophageal adenocarcinoma (EAC). [file 12885_2026_15600_MOESM1_ESM.zip › Supplement 11 EAC.tif]

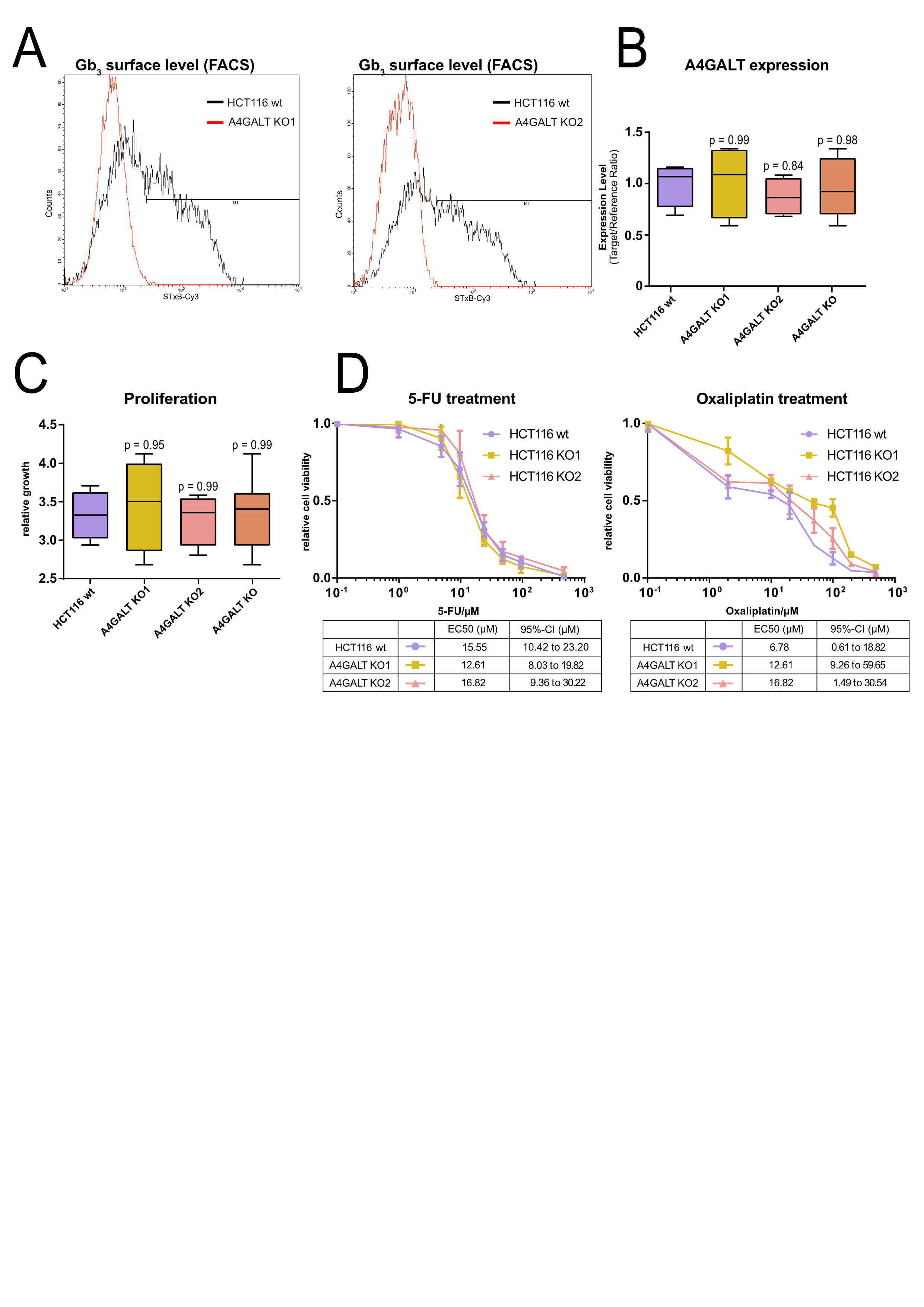

Supplement: Supplementary file 1 — Supplementary Material 1: Supplementary Fig. 1: Genetic alterations in TCGA patients and A4GALT accessibility in DLD1 and HCT116. Supplementary Fig. 2: Generation of A4GALT deficiency. Supplementary Fig. 3: Direct quantification of Gb3 and other lipid species by MALDI2 mass spectrometry. Supplementary Fig. 4: Detailed Gene expression analysis and signatures. Supplementary Fig. 5: Methylation levels. Supplementary Fig. 6: Patient-derived Organoids. Supplementary Fig. 7: Kaplan-Meier survival analysis details. Supplementary Fig. 8: Disease free survival . Supplementary Fig. 9: Progression free survival. Supplementary Fig. 10: Single cell RNAseq analysis. Supplementary Fig. 11: Survival analysis for esophageal adenocarcinoma (EAC). [file 12885_2026_15600_MOESM1_ESM.zip › Supplement 2.tiff]

MALDI2 mass spectrometry

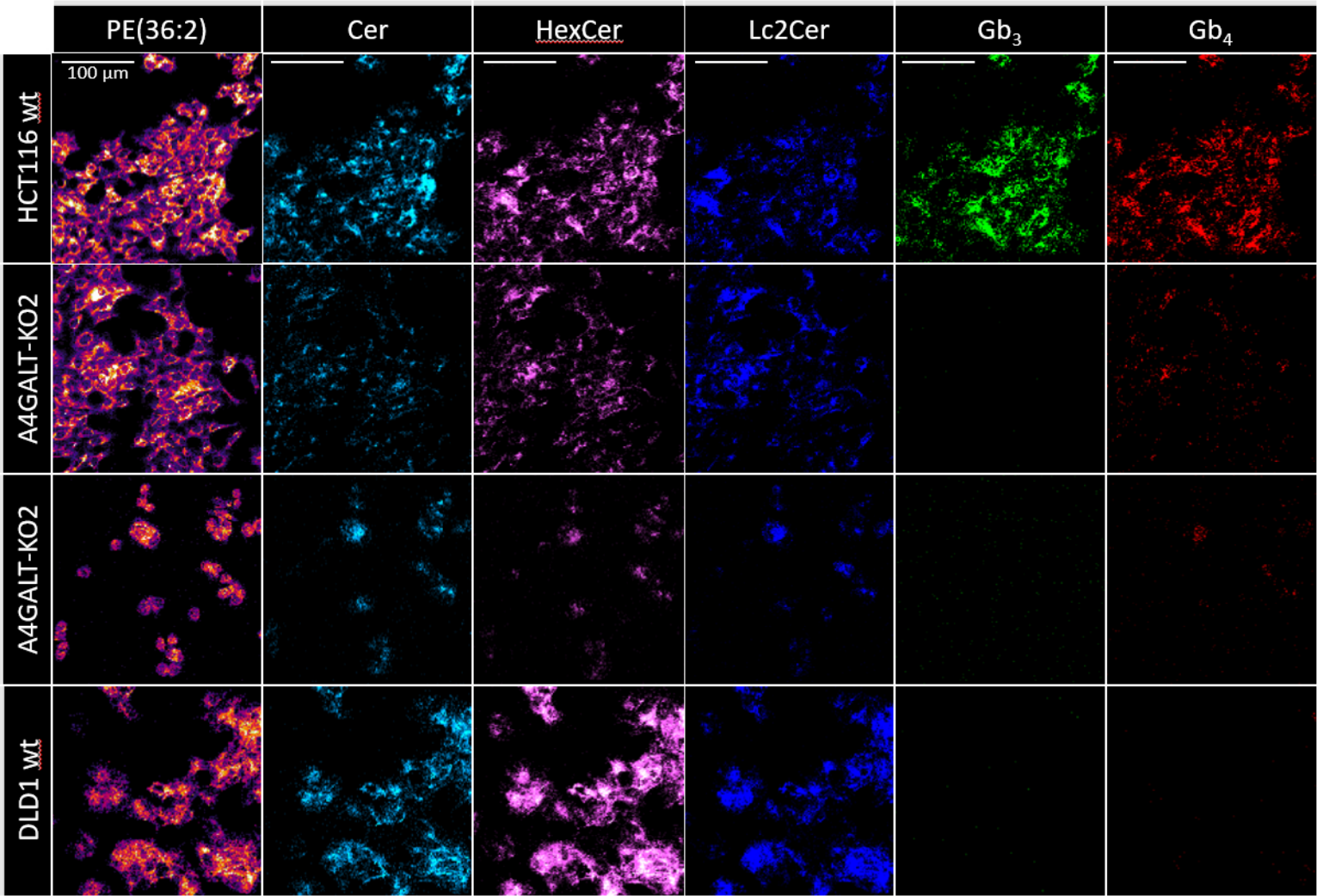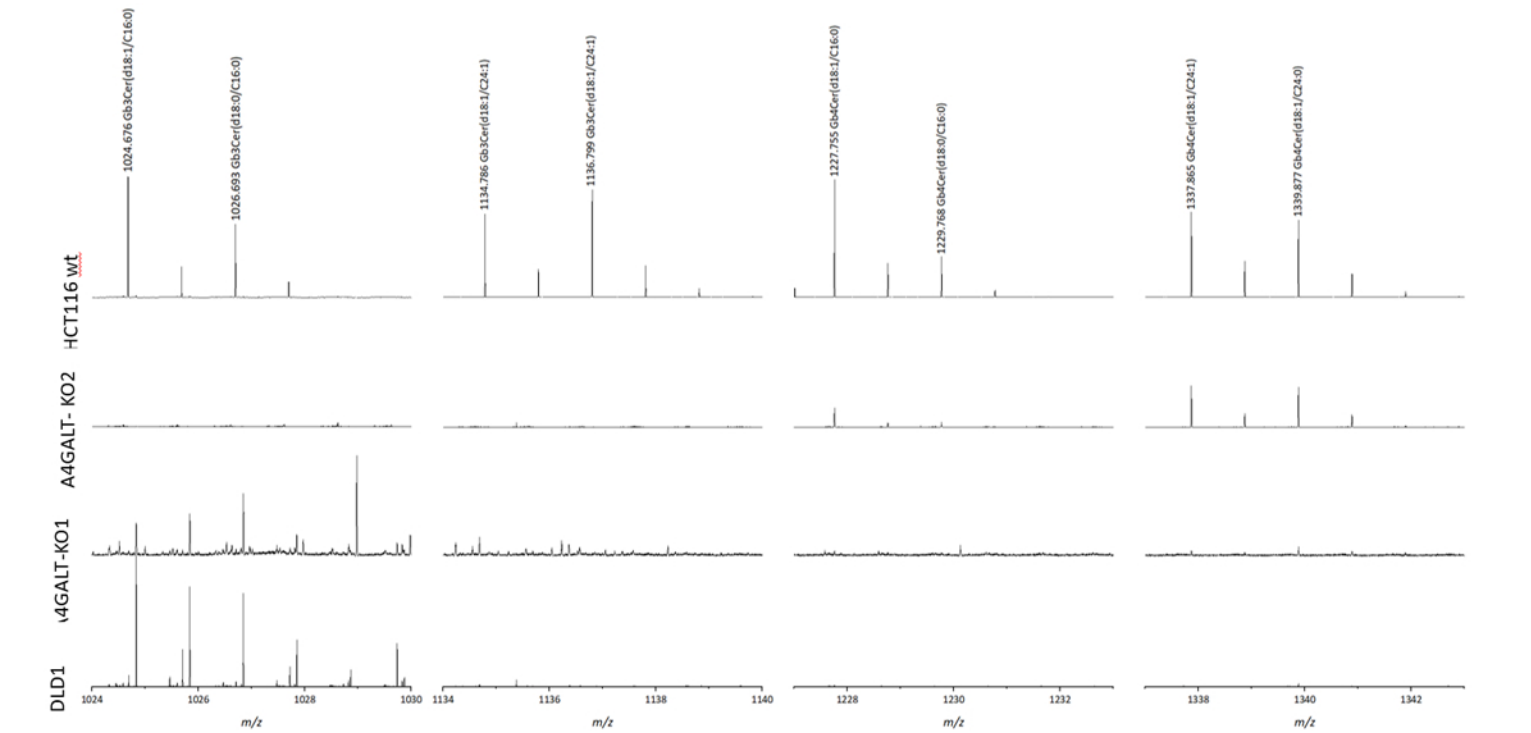

Supplement: Supplementary file 1 — Supplementary Material 1: Supplementary Fig. 1: Genetic alterations in TCGA patients and A4GALT accessibility in DLD1 and HCT116. Supplementary Fig. 2: Generation of A4GALT deficiency. Supplementary Fig. 3: Direct quantification of Gb3 and other lipid species by MALDI2 mass spectrometry. Supplementary Fig. 4: Detailed Gene expression analysis and signatures. Supplementary Fig. 5: Methylation levels. Supplementary Fig. 6: Patient-derived Organoids. Supplementary Fig. 7: Kaplan-Meier survival analysis details. Supplementary Fig. 8: Disease free survival . Supplementary Fig. 9: Progression free survival. Supplementary Fig. 10: Single cell RNAseq analysis. Supplementary Fig. 11: Survival analysis for esophageal adenocarcinoma (EAC). [file 12885_2026_15600_MOESM1_ESM.zip › Supplement 3.pdf]

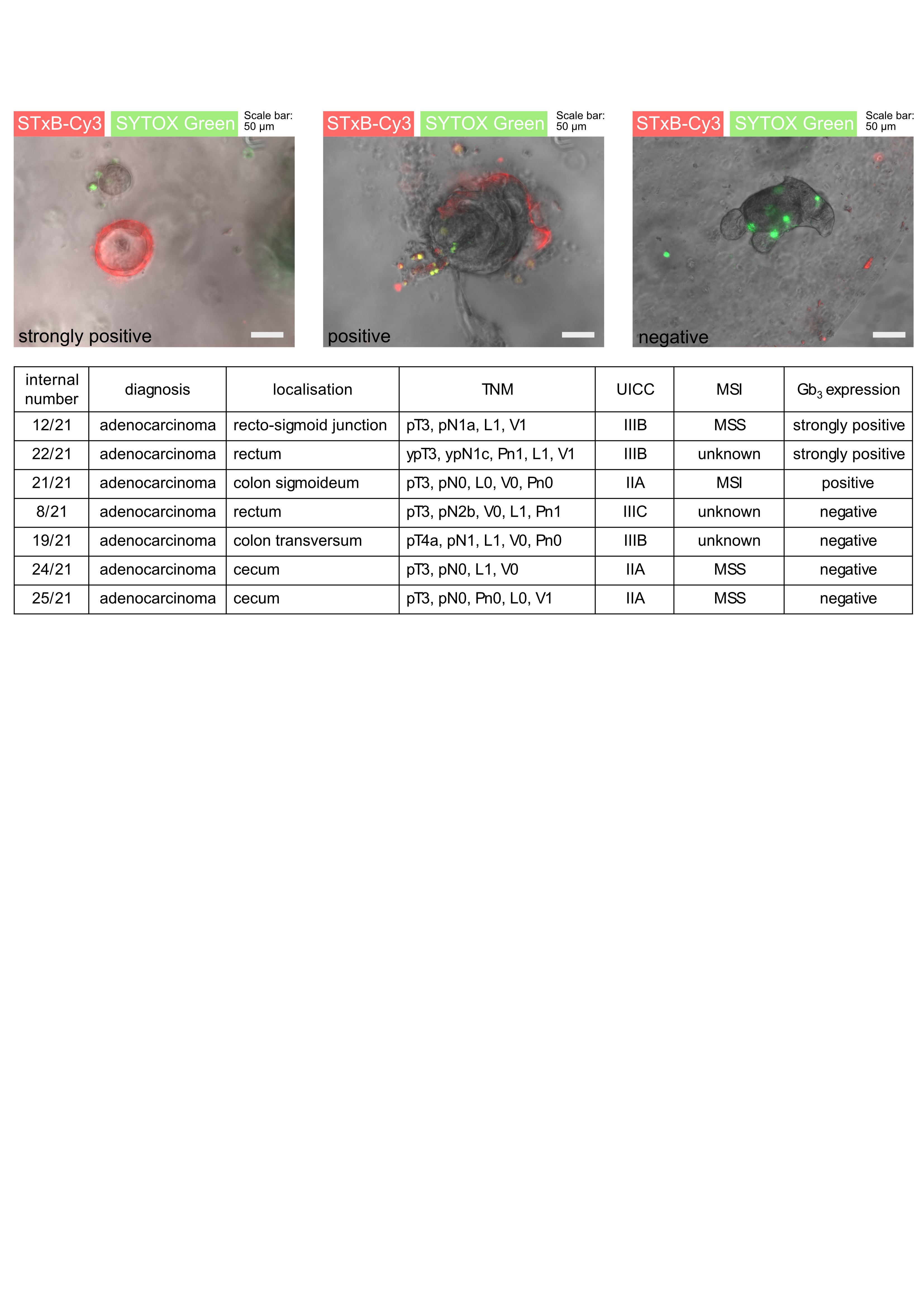

Supplement: Supplementary file 1 — Supplementary Material 1: Supplementary Fig. 1: Genetic alterations in TCGA patients and A4GALT accessibility in DLD1 and HCT116. Supplementary Fig. 2: Generation of A4GALT deficiency. Supplementary Fig. 3: Direct quantification of Gb3 and other lipid species by MALDI2 mass spectrometry. Supplementary Fig. 4: Detailed Gene expression analysis and signatures. Supplementary Fig. 5: Methylation levels. Supplementary Fig. 6: Patient-derived Organoids. Supplementary Fig. 7: Kaplan-Meier survival analysis details. Supplementary Fig. 8: Disease free survival . Supplementary Fig. 9: Progression free survival. Supplementary Fig. 10: Single cell RNAseq analysis. Supplementary Fig. 11: Survival analysis for esophageal adenocarcinoma (EAC). [file 12885_2026_15600_MOESM1_ESM.zip › Supplement 6.tiff]

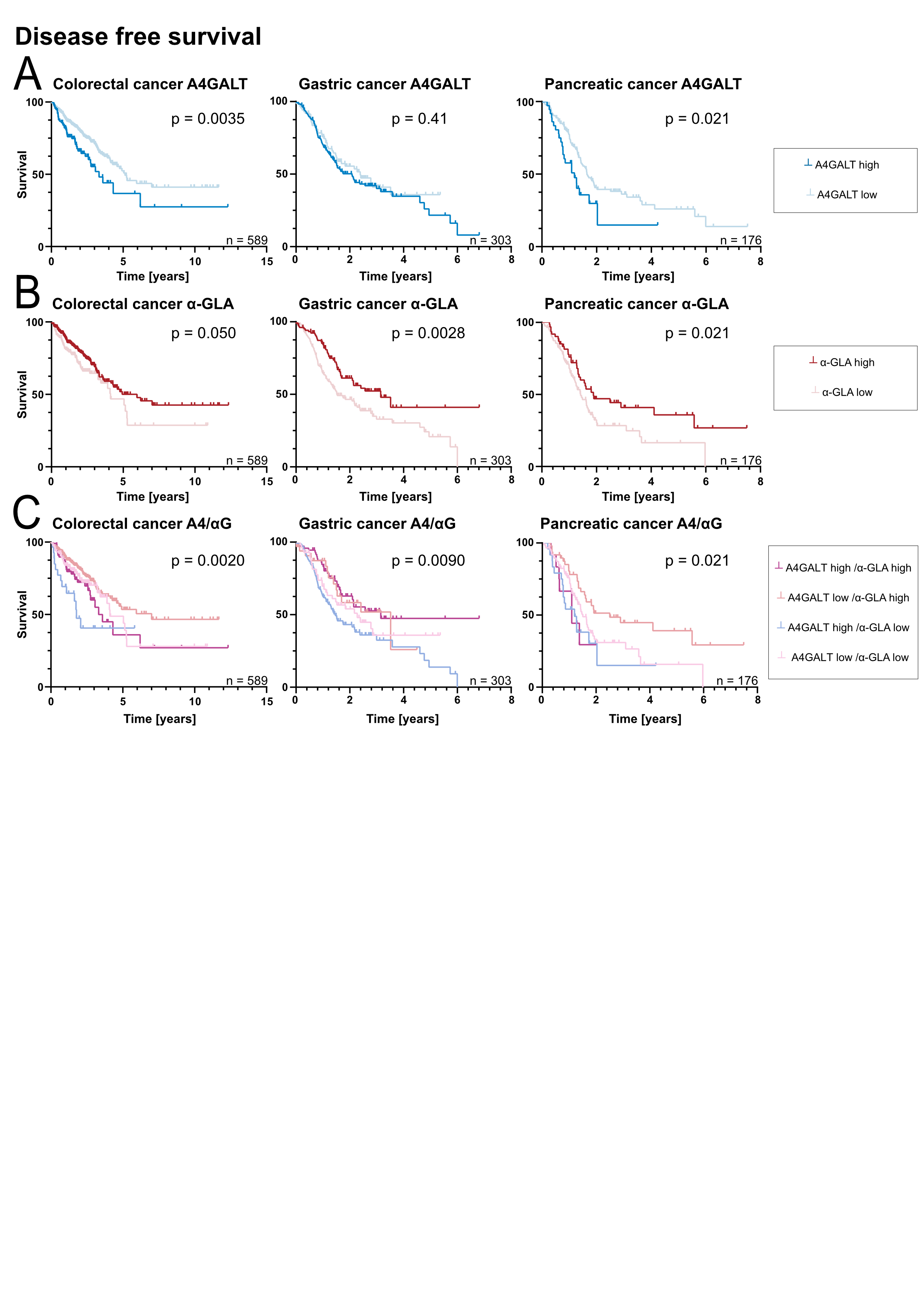

Supplement: Supplementary file 1 — Supplementary Material 1: Supplementary Fig. 1: Genetic alterations in TCGA patients and A4GALT accessibility in DLD1 and HCT116. Supplementary Fig. 2: Generation of A4GALT deficiency. Supplementary Fig. 3: Direct quantification of Gb3 and other lipid species by MALDI2 mass spectrometry. Supplementary Fig. 4: Detailed Gene expression analysis and signatures. Supplementary Fig. 5: Methylation levels. Supplementary Fig. 6: Patient-derived Organoids. Supplementary Fig. 7: Kaplan-Meier survival analysis details. Supplementary Fig. 8: Disease free survival . Supplementary Fig. 9: Progression free survival. Supplementary Fig. 10: Single cell RNAseq analysis. Supplementary Fig. 11: Survival analysis for esophageal adenocarcinoma (EAC). [file 12885_2026_15600_MOESM1_ESM.zip › Supplement 8 DFS.tiff]

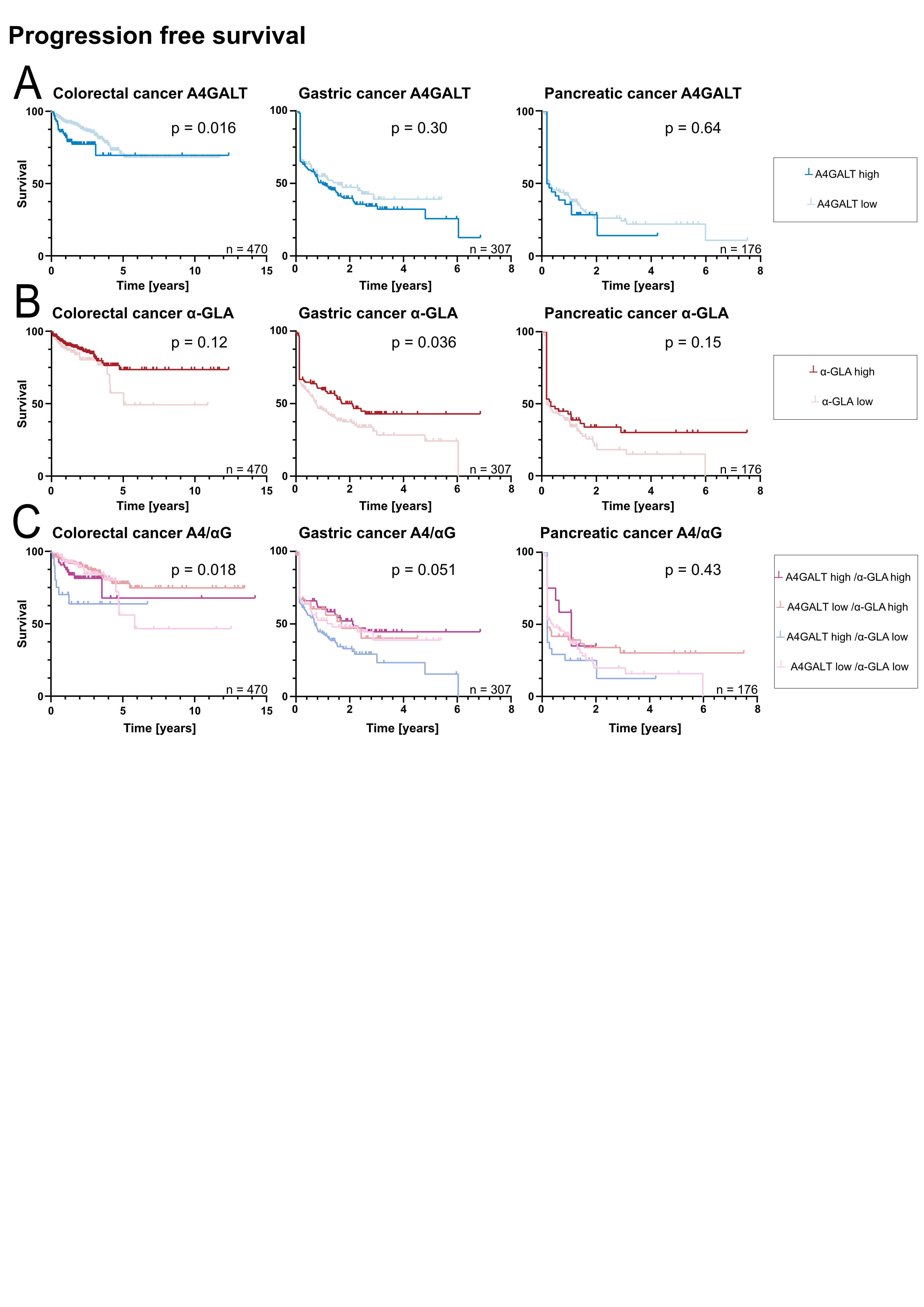

Supplement: Supplementary file 1 — Supplementary Material 1: Supplementary Fig. 1: Genetic alterations in TCGA patients and A4GALT accessibility in DLD1 and HCT116. Supplementary Fig. 2: Generation of A4GALT deficiency. Supplementary Fig. 3: Direct quantification of Gb3 and other lipid species by MALDI2 mass spectrometry. Supplementary Fig. 4: Detailed Gene expression analysis and signatures. Supplementary Fig. 5: Methylation levels. Supplementary Fig. 6: Patient-derived Organoids. Supplementary Fig. 7: Kaplan-Meier survival analysis details. Supplementary Fig. 8: Disease free survival . Supplementary Fig. 9: Progression free survival. Supplementary Fig. 10: Single cell RNAseq analysis. Supplementary Fig. 11: Survival analysis for esophageal adenocarcinoma (EAC). [file 12885_2026_15600_MOESM1_ESM.zip › Supplement 9 PFS.tiff]

# Human colon cancer: single cell RNAseq atlas (c295)

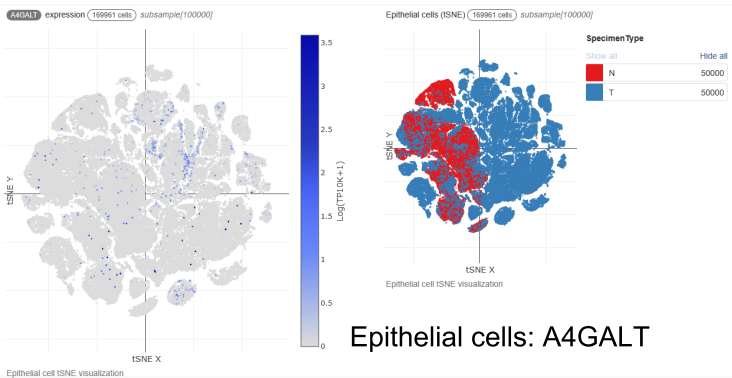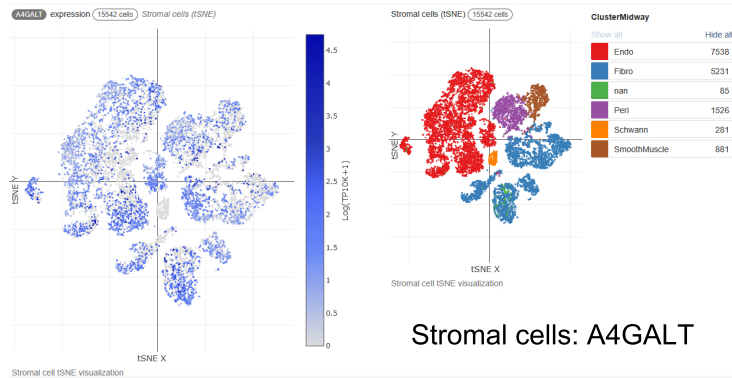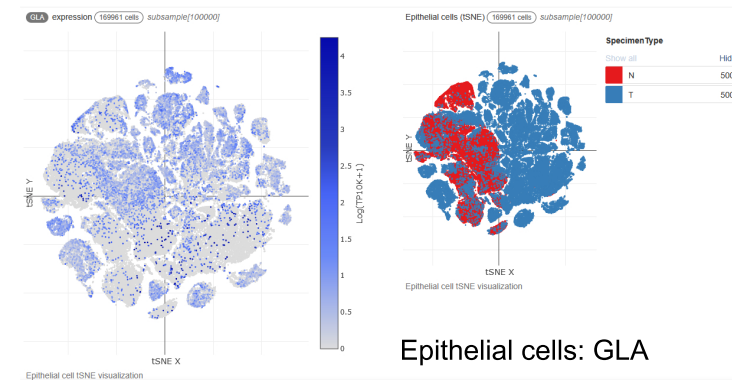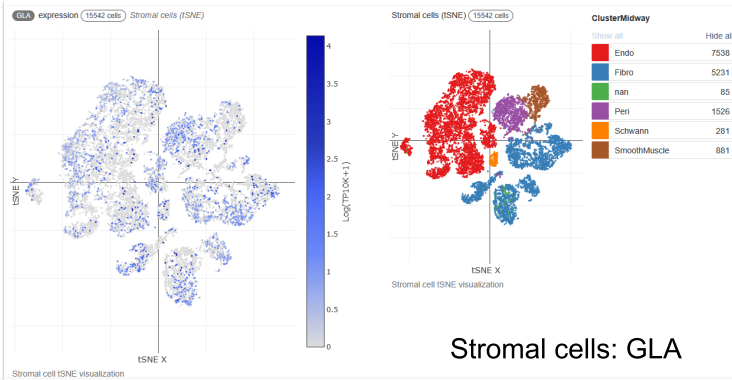

Supplement: Supplementary file 1 — Supplementary Material 1: Supplementary Fig. 1: Genetic alterations in TCGA patients and A4GALT accessibility in DLD1 and HCT116. Supplementary Fig. 2: Generation of A4GALT deficiency. Supplementary Fig. 3: Direct quantification of Gb3 and other lipid species by MALDI2 mass spectrometry. Supplementary Fig. 4: Detailed Gene expression analysis and signatures. Supplementary Fig. 5: Methylation levels. Supplementary Fig. 6: Patient-derived Organoids. Supplementary Fig. 7: Kaplan-Meier survival analysis details. Supplementary Fig. 8: Disease free survival . Supplementary Fig. 9: Progression free survival. Supplementary Fig. 10: Single cell RNAseq analysis. Supplementary Fig. 11: Survival analysis for esophageal adenocarcinoma (EAC). [file 12885_2026_15600_MOESM1_ESM.zip › Supplementary 10 ScRNAseq.pdf]

# Enrichment of Hallmarks in all of aGLA+ Patients

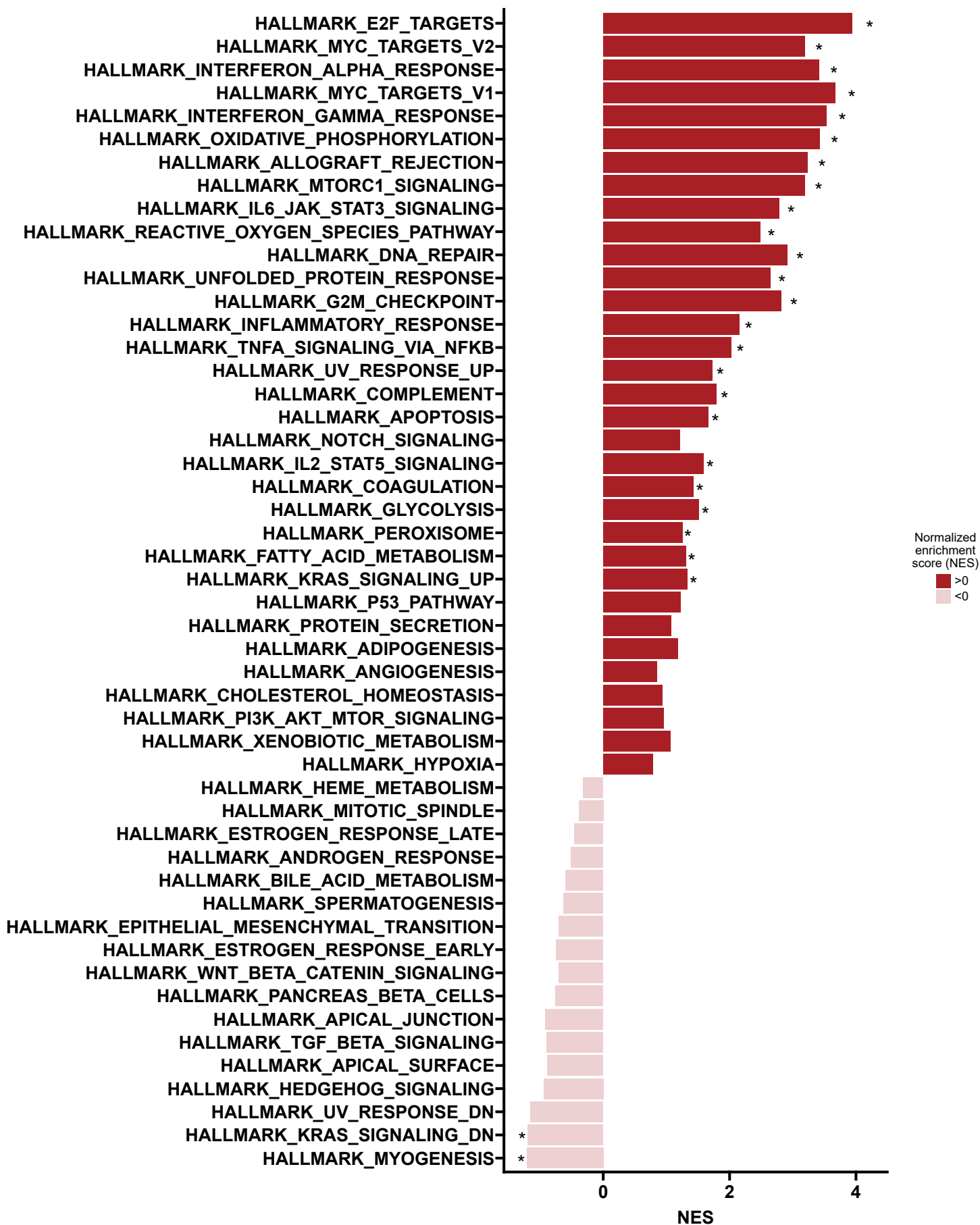

Supplement: Supplementary file 1 — Supplementary Material 1: Supplementary Fig. 1: Genetic alterations in TCGA patients and A4GALT accessibility in DLD1 and HCT116. Supplementary Fig. 2: Generation of A4GALT deficiency. Supplementary Fig. 3: Direct quantification of Gb3 and other lipid species by MALDI2 mass spectrometry. Supplementary Fig. 4: Detailed Gene expression analysis and signatures. Supplementary Fig. 5: Methylation levels. Supplementary Fig. 6: Patient-derived Organoids. Supplementary Fig. 7: Kaplan-Meier survival analysis details. Supplementary Fig. 8: Disease free survival . Supplementary Fig. 9: Progression free survival. Supplementary Fig. 10: Single cell RNAseq analysis. Supplementary Fig. 11: Survival analysis for esophageal adenocarcinoma (EAC). [file 12885_2026_15600_MOESM1_ESM.zip › Supplementary 4a Patients_all_Hallmarks_aGLA.pdf]

# Enrichment of Hallmarks in all of A4GALT+ Patients

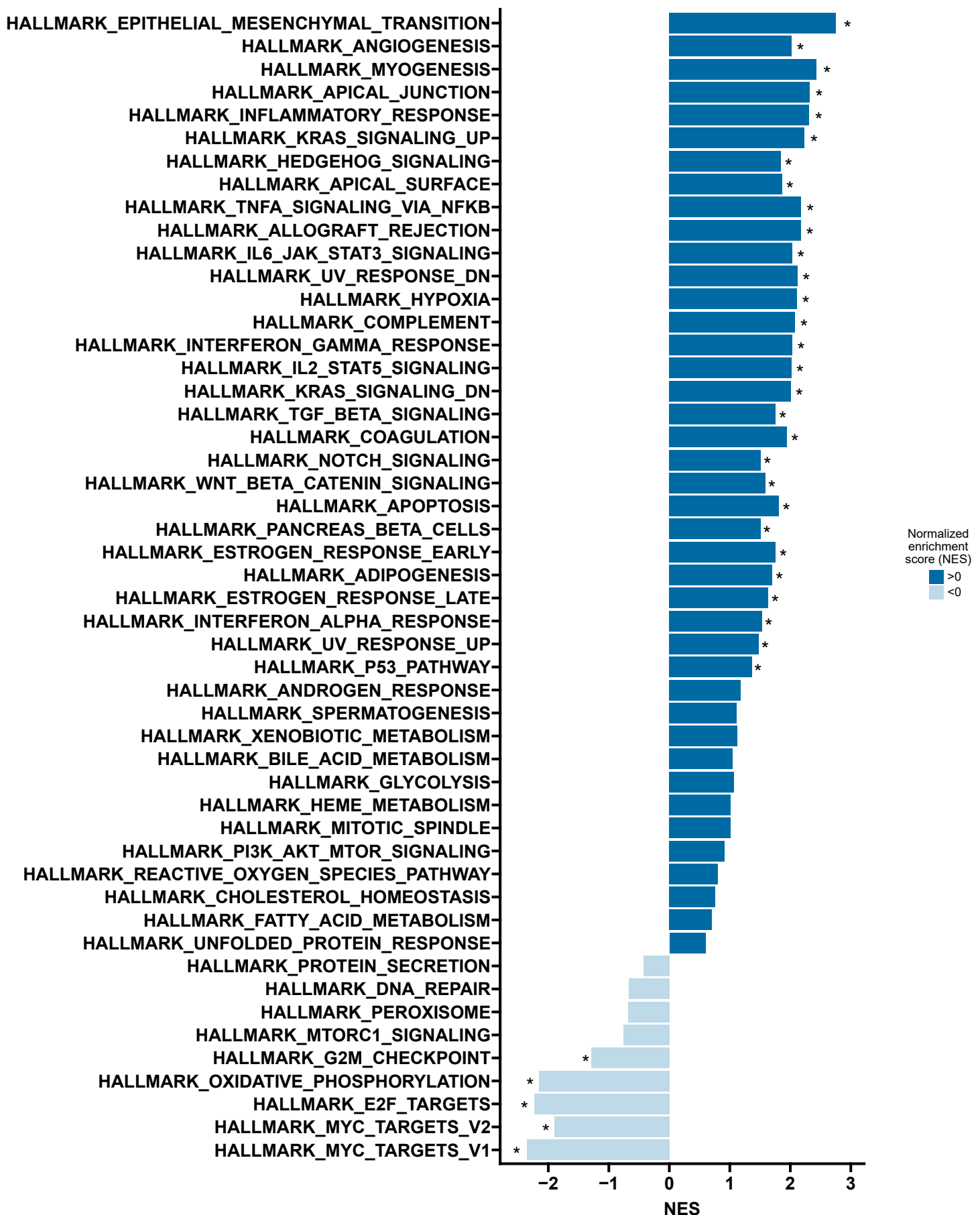

Supplement: Supplementary file 1 — Supplementary Material 1: Supplementary Fig. 1: Genetic alterations in TCGA patients and A4GALT accessibility in DLD1 and HCT116. Supplementary Fig. 2: Generation of A4GALT deficiency. Supplementary Fig. 3: Direct quantification of Gb3 and other lipid species by MALDI2 mass spectrometry. Supplementary Fig. 4: Detailed Gene expression analysis and signatures. Supplementary Fig. 5: Methylation levels. Supplementary Fig. 6: Patient-derived Organoids. Supplementary Fig. 7: Kaplan-Meier survival analysis details. Supplementary Fig. 8: Disease free survival . Supplementary Fig. 9: Progression free survival. Supplementary Fig. 10: Single cell RNAseq analysis. Supplementary Fig. 11: Survival analysis for esophageal adenocarcinoma (EAC). [file 12885_2026_15600_MOESM1_ESM.zip › Supplementary 4a_Patients_all_Hallmarks_A4GALT-2.pdf]
