# Supplementary material for: The Gb3-synthase A4GALT is an epigenetically regulated driver of tumor invasiveness in gastrointestinal cancer
Source: BMC Cancer. 2026 Jan 27;26:274. doi: 10.1186/s12885-026-15600-7 (PMC12917973; doi:10.1186/s12885-026-15600-7)
Supplement: Supplementary file 10 [file 12885_2026_15600_MOESM1_ESM.zip › Figure Legends Supplement.odt]

The Gb3-synthase A4GALT is an epigenetically regulated driver of tumor invasiveness in gastrointestinal cancer

Authors: Noah-David Hirsch, Markus Perl, Simon Holzinger, Christoph Barz, Stefan Enßle, Widya Johannes, Anja Conrad, Jonas J. Unterholzner, Viktoria Obermeier, Markus Tschurtschenthaler, Ludger Johannes, and Klaus-Peter Janssen

# Supplementary Figure Legends

Supplementary Fig. 1: Genetic alterations in TCGA patients and A4GALT accessibility in DLD1 and HCT116

a Genetic alterations in TCGA patients with colorectal cancer (CRC), gastric cancer (GC), and pancreatic cancer (PC), each stratified by high (+) and low (-) A4GALT expression. Except for a small number of patients (CRC A4GALT- and GC A4GALT+), no genetic alterations were observed. 

b Cumulative chromatin accessibility (ATAC-seq) at the internal regulatory region of the A4GALT locus in DLD1 (light blue) and HCT116 (dark blue) cells.

Supplementary Fig. 2: Generation of A4GALT deficiency 

a Gb3 surface levels measured by flow cytometry (FACS) in A4GALT knockout clones in the cell line HCT116 (red; A4GALT-KO1 left, A4GALT-KO2 right) showed a marked reduction compared with wild-type cells (black). The M1 gate indicates the threshold of signal intensity used to distinguish Gb3-positive from -negative cells.
b A4GALT mRNA expression assessed by rt-qPCR in A4GALT knockout clones showed no significant differences compared with wild-type cells (presented as mean ± SD; n ≥ 3).
c Proliferation rates of A4GALT knockout clones, measured by XTT assays, showed no significant differences compared with wild-type cells (presented as mean ± SD; n ≥ 3).
d Dose–response curves for 5-fluorouracil and oxaliplatin treatment measured by XTT assays. EC50 values of A4GALT knockout clones showed no significant differences compared with wild-type cells (presented as mean ± SD; n ≥ 3).

Supplementary Fig. 3: Direct quantification of Gb3 and other lipid species by MALDI2 mass spectrometry. 

Analysis by MALDI2 corroborated the findings by indirect Gb3-staining by STxB for both A4GALT-deficient lines, in comparison to parental HCT116 cells and DLD1 cells (negative for Gb3). Left to right: As technical control, the phospholipid phosphatidylethanolamine (PE 36:2) was detected in all cell lines. The precusor lipid species ceramide (Cer), as starting point for all glycosphingolipids of the globo-series, as well as glucosylceramide (Hexosylceramide, HexCer) and lactosylceramide (Lc2Cr), were detectable in both A4GALT-KO lines and in DLD1 cells, at similar levels as in parental HCT116 cells. Essentially no Gb3 or Gb4 (globotetraosylceramide) was detectable in both KO lines, as well as in DLD1 cells, which do not express A4GALT. Size bars: 100 µM. Lower panels: annotated MALDI-2 mass spectra acquired from the cell line samples displayed above, with various Gb3 lipoforms consistently lacking in A4GALT deficient clones and in DLD1 cells. All annotated ions represent [M + H]+ species. Spectra display the sum over a few dozen pixels from areas high in the certain analyte. Signals were assigned based on accurate mass using a ≤ 1 ppm criterion. Supported by the MS/MS data from selected precursors, all GSL are assumed to comprise a d18:1 sphingosine. MS data courtesy of Dr. T. Bien and Prof. K. Dreisewerd.

Supplementary Fig. 4: Detailed Gene expression analysis and signatures

Gene expression datasets as tables (.tsv format), volcano plots, and complete Hallmark pathway enrichment figures, shown for A4GALT and GLA, respectively, grouped for: 

a all patients

b colorectal carcinoma

c gastric carcinoma

d pancreatic carcinoma

Supplementary Fig. 5: Methylation levels 

DNA methylation of the analyzed CpG islands (Illumina 450k methylation array) at the A4GALT locus in CRC (top), GC (middle), and GC (bottom) patients with high (dark blue) or low (light blue) A4GALT expression. P values were adjusted for multiple testing using the false discovery rate (fdr).

Supplementary Fig. 6: Patient-derived Organoids

CRC organoids were screened for Gb3 expression. Representative immunofluorescence images (top) show CRC organoids stained with STxB-Cy3 (red, Gb3) and SYTOX Green (green). Scale bar: 100 µm. Gb3 expression was classified as strongly positive (left), positive (middle), or negative (right). In total, seven tumor organoid lines were analyzed. Gb3 expression levels for each line, along with their clinical characteristics, are summarized in the table below.

Supplementary Fig. 7: Kaplan-Meier survival analysis details

(A) Baseline characteristics of the TCGA patient cohorts

(B) Kaplan-Meier survival analysis of stratification by A4GALT and α-GLA expression only showing sizes, number of deaths, number of censored, median survival and hazard ratios.

(C) Kaplan-Meier survival analysis of combined expression analysis of A4GALT and α-GLA (A4GALT+/α-GLA+, A4GALT+/α-GLA-, A4GALT-/α-GLA+, A4GALT-/α-GLA-) showing sizes, number of deaths, number of censored, median survival and hazard ratios.

Supplementary Fig. 8: Disease free survival 

(A) Kaplan-Meier survival curves for patients with colorectal carcinoma (CRC, n = 589), gastric carcinoma (GC, n = 303), and pancreatic carcinoma (PC, n = 176) stratified into high and low expression groups for A4GALT. Low A4GALT expression was significantly associated with improved disease-free survival in CRC (p = 0.0035) and PC (p = 0.021), but not in GC (p = 0.41).

(B) Kaplan-Meier survival curves based on α-GLA expression in the same cancer types and cohorts. High α-GLA expression conferred a significant or highly significant disease-free survival advantage (CRC: p = 0.050; GC: p = 0.0028; PC: p = 0.021).

(C) Combined expression analysis of A4GALT and α-GLA (A4GALT+/α-GLA+, A4GALT+/α-GLA-, A4GALT-/α-GLA+, A4GALT-/α-GLA-) in CRC, GC, and PC. Significant differences in disease-free survival were present in all cancer entities (CRC: p = 0.0020; GC: p = 0.0090; PC: p = 0.021). 

Supplementary Fig. 9: Progression free survival

(A) Kaplan-Meier survival curves for patients with colorectal carcinoma (CRC, n = 470), gastric carcinoma (GC, n = 307), and pancreatic carcinoma (PC, n = 176) stratified into high and low expression groups for A4GALT. Low A4GALT expression was significantly associated with improved progression free survival in CRC (p = 0.0016), but not in PC (p = 0.64) and GC (p = 0.30).

(B) Kaplan-Meier survival curves based on α-GLA expression in the same cancer types and cohorts. High α-GLA expression conferred a significant progression free survival advantage in GC (p = 0.036) and a non-significant trend in CRC (p = 0.12) and PC (p = 0.15).

(C) Combined expression analysis of A4GALT and α-GLA (A4GALT+/α-GLA+, A4GALT+/α-GLA-, A4GALT-/α-GLA+, A4GALT-/α-GLA-) in CRC, GC, and PC. Significant differences in progression free survival were only present in CRC (p = 0.018; GC: p = 0.051; PC: p = 0.43). 

Supplementary Fig. 10: Single cell RNAseq analysis. A public ScRNAseq dataset of human colorectal cancer (Broad Institute, Human Colon Cancer Atlas doi: https://doi.org/10.1101/2023.07.13.548886) was analyzed for A4GALT and GLA expression. Even though A4GALT was generally expressed at low levels only, it was found in tumor cells of epithelial origin, as well as in stroma cells, but essentially not detectable in normal epithelia. GLA in contrast was more broadly expressed and also found in normal epithelial cells. 

Supplementary Fig. 11: Survival analysis for esophageal adenocarcinoma (EAC)

(A) Kaplan-Meier survival curves for patients with esophageal adenocarcinoma (EAC) stratified into high and low expression groups for A4GALT and analyzed for overall survival (OS, n = 86), progression free survival (PFS, n = 86) and disease-free survival (DFS, n = 86). High A4GALT expression was associated with improved overall survival (p = 0.024), disease-free survival (p = 0.006) and progression-free survival (p = 0.013).

(B) Kaplan-Meier survival curves based on α-GLA expression in the same cohorts and survival endpoints. Low α-GLA expression conferred a significant advantage for all survival endpoints (OS: p = 0.0003; PFS: p = 0.0018; DFS: p = 0.0008).

(C) Combined expression analysis of A4GALT and α-GLA (A4GALT+/α-GLA+, A4GALT+/α-GLA-, A4GALT-/α-GLA+, A4GALT-/α-GLA-) in the same cohorts and survival endpoints. Significant differences were present for all survival endpoints (OS: p = 0.0006; PFS: p = 0.0023; DFS: p = 0.0014). Lower panels: Gb3 levels were tested in vitro in  three cell lines derived from human esophageal cancer (OE19, OE21 and OE33), and the gastric cancer line St3051 (positive control), by FACS after staining with STxB-Cy3, as described in the main text for HCT116 cells. Expression of Gb3 was low to moderate in OE19, the other two lines were essentially negative for Gb3.
